# Theta burst stimulation for depression: a systematic review and network and pairwise meta-analysis

**DOI:** 10.1038/s41380-024-02630-5

**Published:** 2024-06-06

**Authors:** Taro Kishi, Toshikazu Ikuta, Kenji Sakuma, Masakazu Hatano, Yuki Matsuda, Jonas Wilkening, Roberto Goya-Maldonado, Martin Tik, Nolan R. Williams, Shinsuke Kito, Nakao Iwata

**Affiliations:** 1 Department of Psychiatry, Fujita Health University School of Medicine, Toyoake, Aichi 470-1192 Japan; 2 Department of Communication Sciences and Disorders, School of Applied Sciences, University of Mississippi, University, MS 38677 USA; 3 Department of Pharmacotherapeutics and Informatics, Fujita Health University School of Medicine, Toyoake, Aichi 470-1192 Japan; 4 Department of Development and Education of Clinical Research, Fujita Health University School of Medicine, Toyoake, Aichi 470-1192 Japan; 5 Department of Psychiatry, Jikei University School of Medicine, Minato-ku, Tokyo 105-8461 Japan; 6 Laboratory of Systems Neuroscience and Imaging in Psychiatry (SNIP-Lab), Department of Psychiatry and Psychotherapy, University Medical Center Göttingen (UMG), Göttingen, 37075 Germany; 7 Department of Psychiatry and Behavioral Sciences, Stanford University, Stanford, CA 94305 USA

**Keywords:** Depression, Bipolar disorder

## Abstract

In clinical practice, theta burst stimulation (TBS) presents as a more efficient and potentially more effective therapeutic modality than conventional repetitive transcranial magnetic stimulation (rTMS), as it allows for the delivery of more stimuli in less time and at similar intensities. To date, accelerated treatment plans according to various continuous (cTBS) and intermittent TBS (iTBS) protocols for depression have been proposed. To investigate which of the TBS protocols provided a favorable risk-benefit balance for individuals with depression, this systematic review and random-effects model network meta-analysis was conducted. The study outcomes included response rate (primary), depression symptom improvement, remission rate, all-cause discontinuation rate, incidence of switch to mania, and incidence of headache/discomfort at treatment site. In this meta-analysis, a total of 23 randomized controlled trials (*n* = 960, mean age = 41.88 years, with 60.78% females) were included. Approximately 69.57% of the trials included individuals with an exclusive diagnosis of major depressive disorder. The following six TBS protocols (target) were evaluated: cTBS (right-dorsolateral prefrontal cortex [R-DLPFC]), cTBS (R-DLPFC) + iTBS (left-DLPFC [L-DLPFC]), iTBS (L-DLPFC), iTBS (L-DLPFC) + iTBS (R-DLPFC), iTBS (left-dorsomedial prefrontal cortex) + iTBS (right-dorsomedial prefrontal cortex), and iTBS (occipital lobe). Compared to sham, cTBS (R-DLPFC) + iTBS (L-DLPFC), iTBS (L-DLPFC), and iTBS (occipital lobe) had a higher response rate (*k* = 23); cTBS (R-DLPFC) + iTBS (L-DLPFC) and iTBS (L-DLPFC) dominated in the depression symptom improvement (*k* = 23); and iTBS (L-DLPFC) had a higher remission rate (*k* = 15). No significant differences were found for all-cause discontinuation rate (*k* = 17), incidence of switch to mania (*k* = 7), and incidence of headache/discomfort at treatment site (*k* = 10) between any TBS protocols and sham. Thus, cTBS (R-DLPFC) + iTBS (L-DLPFC) and iTBS (L-DLPFC) demonstrate favorable risk-benefit balance for the treatment of depression.

## Introduction

Repetitive transcranial magnetic stimulation (rTMS) is recommended as treatment for individuals with pharmacological treatment-resistant depression (TRD) according to evidence [1–3]. The most common method of rTMS therapy includes placing a treatment coil on the patient’s scalp over the frontal lobe that generates electromagnetic pulses, which induce electric fields modulating the targeted left dorsolateral prefrontal cortex (L-DLPFC) and functionally connected networks [4–6]. The conventional treatment plans involve high-frequency ( ~ 10 Hz) rTMS (HF-rTMS) targeting L-DLPFC (HF-rTMS [L-DLPFC]). However, as treatment sessions last approximately 40 min and the initial course of treatment typically consists of five treatments per week over a 6-week period, the daily application over the course of multiple weeks and the delayed time-to-response limit its practicality for both patient and treatment clinics [7, 8]. In 2018, the US Food and Drug Administration (FDA) approved intermittent theta burst stimulation (iTBS) using 600 pulses targeting L-DLPFC (iTBS [L-DLPFC]) as a treatment for depression based on the evidence of noninferiority over conventional HF-rTMS (L-DLPFC) in a large multi-center trial [8]. While conventional HF-rTMS (L-DLPFC) treatment takes approximately 40 min per session, the similar effective dose of TMS with iTBS (L-DLPFC) can be delivered in just 3 min.

In a previous pairwise meta-analysis [9], we showed that iTBS (L-DLPFC) and HF-rTMS (L-DLPFC) demonstrate no significant differences in efficacy, acceptability, and safety profiles. Thus, iTBS (L-DLPFC) could be offered as a more practical and potentially more efficient therapeutic modality clinically. However, the FDA-approved single daily iTBS (L-DLPFC) course still requires a considerable 6-week treatment duration of five daily treatments. Accelerated iTBS protocols have been proposed to address this issue, delivering multiple iTBS sessions per day to deliver a number of pulses within shorter timeframes. These accelerated protocols not only have the potential to treat more patients within the same time period but also to reduce patient visit frequency to rTMS clinics, thereby enhancing overall accessibility and treatment adherence [10].

To date, the most accelerated iTBS (L-DLPFC) treatment protocol has been the Stanford Neuromodulation Therapy (SNT) [11]. This protocol comprises 10 sessions of iTBS (L-DLPFC) that are delivered daily, for a total of 18,000 pulses per day (i.e., total number of pulses of conventional iTBS 6-week protocol) on five consecutive days [11]. A randomized controlled trial (RCT) of SNT demonstrated that iTBS (L-DLPFC) outperformed sham in depressive symptom improvement with a large effect size (Cohen’s *d* > 0.8) [11].

To date, in addition to iTBS (L-DLPFC), numerous other theta burst stimulation (TBS) protocols such as continuous TBS (cTBS) over the right DLPFC (R-DLPFC) (cTBS [R-DLPFC]) have been proposed (Table S1). Thus, this systematic review and random-effects model network meta-analysis was conducted to investigate, which TBS protocols produced favorable risk-benefit balance for individuals with depression. In the current meta-regression analyses, since potential modifiers associated with TBS efficacy in individuals with depression remain unknown, we attempted to identify variables in participants, treatment, and/or study design that influence the effect size for response rate (the primary outcome of the current study). Our meta-regression further aimed at identifying TBS response modifiers and the interplay between these modifiers and the sham response in the formation of effect sizes.

## Methods

This study was conducted in line with the Preferred Reporting Items for Systematic Reviews and Meta-Analyses guidelines (Table S2) [12, 13] and was registered on the Open Science Framework (https://osf.io/m6tf3). The literature search, data transfer accuracy, and calculations were evaluated by at least two authors.

### Search strategy and inclusion criteria

In Fig. S1, detailed information regarding the search strategy is demonstrated. The inclusion criteria for the studies were as follows: (1) published and unpublished RCTs that had at least two TBS treatment sessions, (2) RCTs including individuals with both or either major depressive disorder (MDD) and/or bipolar depression (BDep), (3) RCTs including individuals with TRD and/or individuals with no TRD. The exclusion criteria were as follows: (1) RCTs that included individuals with a dual diagnosis of depression and substance-use disorders and (2) RCTs that included individuals with other psychiatric disorders, such as schizophrenia other than MDD and BDep. We searched PubMed, the Cochrane Library, and Embase databases for studies published prior to August 4, 2023.

### Outcome measures, data synthesis, and data extraction

The current study outcomes included the response rate (primary), depression symptom improvement, remission rate, all-cause discontinuation rate, incidence of switch to mania, and incidence of headache/discomfort at treatment site. In Table S3, the data synthesis for efficacy outcomes is demonstrated. We conducted a meta-analysis for the outcomes, which included at least five RCTs. The extracted data were analyzed according to the intention-to-treat or modified intention-to-treat principles. We searched for the data in published systematic review articles if required data were missing in the studies. We also attempted to contact the original investigators to obtain unpublished data.

### Meta-analysis methods

Both pairwise and frequentist network meta-analyses were performed using the random-effects model [14, 15]. The risk ratio (RR) for dichotomous variables or the standardized mean difference (SMD) for continuous variables was calculated, with 95% confidence intervals (95% CI). Network heterogeneity was assessed using *τ*^2^ statistics. For pairwise meta-analyses, *I*^2^ statistics was utilized to evaluate heterogeneity. Statistical evaluation of incoherence was performed using the design-by-treatment test (globally) [16] and the Separating Indirect from Direct Evidence (SIDE) test (locally) [17]. The surface under the curve cumulative ranking probabilities were used to rank the treatments for each outcome. To validate the analysis, we determined whether the distribution differences were sufficient by comparing the distribution of the possible effect modifiers across treatments included in the network meta-analysis using the Kruskal–Wallis test (continuous variables) and the Pearson chi-squared test or the Fisher exact test (categorical variables) and by evaluating their actual impact on the treatment effect via meta-regression analyses [18–20]. The potential confounding factors were as follows: mean age, female proportion, total number of participants, minimum depressive symptoms at baseline, diagnostic classification, publication year, overall risk of bias, coil localization/targeting method, and total number of sessions during the study (Table S4). We evaluated overall risk of bias for every RCT using version 2 of the Cochrane risk of bias tool for randomized trials (https://www.riskofbias.info/). Finally, the results were incorporated into the Confidence in Network Meta-Analysis application, an adaptation of the Grading of Recommendations Assessment, Development, and Evaluation approach, to evaluate the credibility of the findings of each of the network meta-analyses [21–23].

For TBS protocols that only outperformed from a sham with respect to primary outcome in our network meta-analysis (i.e., cTBS [R-DLPFC] + iTBS [L-DLPFC] and iTBS [L-DLPFC]), a single-group summary meta-analysis was conducted to identify the exact response rates with 95% CIs in both the TBS and sham groups. We conducted meta-regression analyses to examine whether the differences in the characteristics of the participants, treatment, and/or study design influenced the effect size of the protocols for the primary outcome. The following moderators were involved: 1. factors related to the participants: (1) diagnosis, (2) TRD, (3) proportion of females, (4) mean age, and (5) total number of participants; 2. factors related to the treatments: (6) coil localization/targeting methods, (7) sham condition use, (8) percent motor threshold, (9) number of sessions during a day, (10) number of sessions during a trial, (11) number of pulses during a session, (12) number of pulses during a day, (13) number of pulses during a trial, (14) accelerated TBS protocol, and (15) intersession interval; 3. factors related to the study design: (16) publication year and (17) overall risk of bias (Appendix S1). Furthermore, the following two sensitivity pairwise meta-analyses were performed: (1) a sensitivity pairwise meta-analysis excluding an RCT [24] including only individuals with mixed episode from the primary pairwise meta-analysis of cTBS (R-DLPFC) + iTBS (L-DLPFC) and (2) another sensitivity pairwise meta-analysis excluding an SNT study [11] from the primary pairwise meta-analysis of iTBS (L-DLPFC). Some differences were observed in the study characteristics between SNT study and others. For example, an interim analysis was used for the SNT study. While this could incite several biases [25], this interim analysis was preplanned by the authors to be conducted if the study showed a large effect size of active compared with sham treatment (Cohen’s *d* > 0.8). The superiority of the protocol could be explained by several factors: First, the SNT study utilized individualized targets derived from repeated runs of high-resolution functional connectivity magnetic resonance imaging. Second, stimulation intensity was adjusted for differences in the target-depth. Third, the hourly administration of the protocol with 50-min intersession interval leads to a cumulative effect, and finally, SNT had a higher total number of pulses than other studies. The Comprehensive Meta-Analysis Software Version 3 (Biostat Inc., Englewood, NJ, USA) was used to conduct a pairwise meta-analysis and meta-regression.

## Results

### Study characteristics

A flowchart of the literature search and a detailed explanation of the process are presented in Fig. S1. A total of 546 articles were initially identified; of these, 146 were duplicates, after title and abstract screening, 366 articles were excluded, and a further 11 were excluded after full-text review. No additional study was found from previous review articles. Finally, a total of 23 RCTs (n = 960, mean age = 41.88 years, with 60.78% females) were included in the meta-analysis [11, 24, 26–46]. Approximately 69.57% trials only included individuals with MDD. We evaluated six TBS protocols (target): cTBS (R-DLPFC), cTBS (R-DLPFC) + iTBS (L-DLPFC), iTBS (L-DLPFC), iTBS (L-DLPFC) + iTBS (R-DLPFC), iTBS (left-dorsomedial prefrontal cortex) + iTBS (right-dorsomedial prefrontal cortex), and iTBS (occipital lobe). The study characteristics are summarized in Table S1. None of the studies were provided with industry sponsorship. In most of the studies, the overall risk of bias was evaluated as “some concerns” (Table S5). Across different comparisons, there was no evidence of transitivity assumption violations when comparing the study characteristics (Table S4).

### Network meta-analysis results

**Fig. 1:**
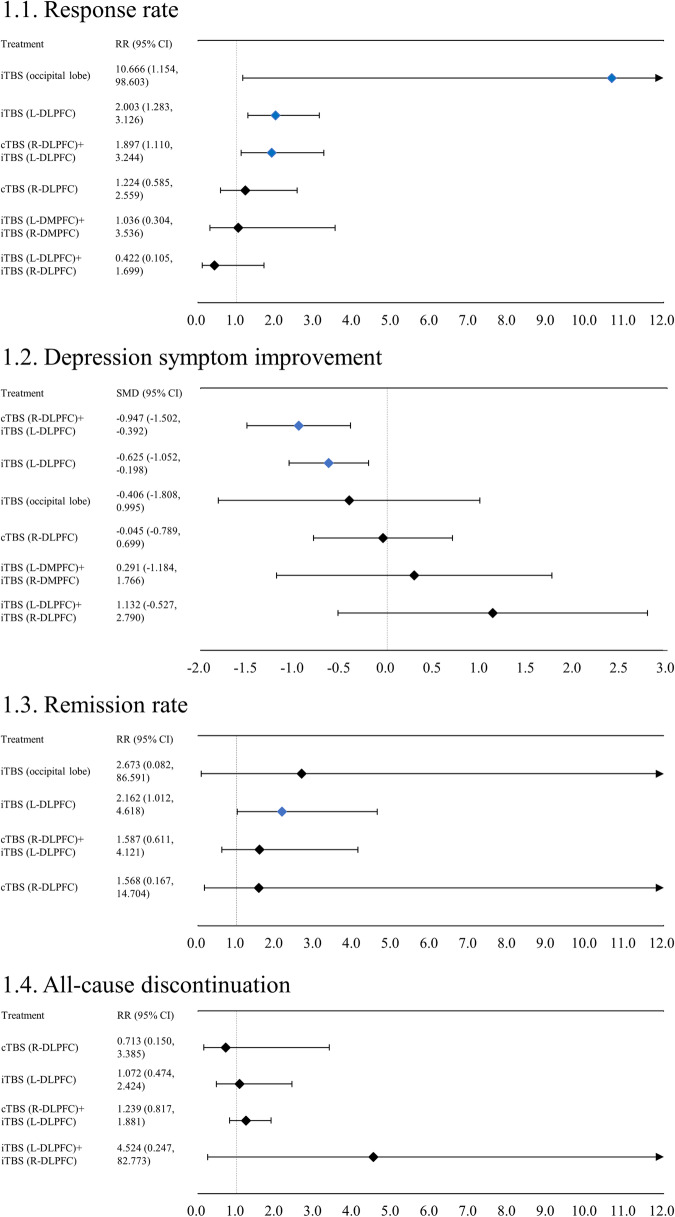
Forest plot. 1.1. Response rate. Values above 1 favor the active treatment. 1.2. Depression symptom improvement. Values below 0 favor the active treatment. 1.3. Remission rate. Values above 1 favor the active treatment. 1.4. All-cause discontinuation. Values below 1 favor the active treatment. 95% CI: 95% confidence interval; cTBS: continuous theta burst stimulation; iTBS: intermittent theta burst stimulation; L (or R-) DLPFC: left (or right-) dorsolateral prefrontal cortex; L (or R-) DMPFC: left (or right-) dorsomedial prefrontal cortex; RR: risk ratio; SMD: standardized mean difference. Active treatments were compared with the sham. Colors indicate the presence or absence of a significant difference: blue, the active treatment was superior to the sham; black, the active treatment was similar to the sham.

cTBS (R-DLPFC) + iTBS (L-DLPFC), iTBS (L-DLPFC), and iTBS (occipital lobe) had a higher response rate compared to the sham (k = 23, Fig. 1, Appendix S1). The RRs (95% CIs) for cTBS (R-DLPFC) + iTBS (L-DLPFC), iTBS (L-DLPFC), and iTBS (occipital lobe) were 1.897 (1.110, 3.244), 2.003 (1.283, 3.126), and 10.666 (1.154, 98.603), respectively. No global consistency was found for the primary outcome, although global heterogeneity was moderate to high. In the meta-analysis results, a significant local inconsistency was found for only iTBS (L-DLPFC) vs. sham regarding the response rate between network meta-analysis (RR [95% CI] = 2.003 [1.283, 3.126]) and pairwise meta-analysis (RR [95% CI] = 2.290 [1.437, 3.649]). No comparisons were included in at least 10 studies other than iTBS (L-DLPFC); however, the funnel plots of the response rates demonstrated symmetry (Appendix S1). No potential confounding factors were associated with the effect size of the primary outcome on the meta-regression analyses (Appendix S1).

Both, cTBS (R-DLPFC) + iTBS (L-DLPFC) and iTBS (L-DLPFC) demonstrated superiority over sham in depression symptom improvement (*k* = 23, Fig. 1, Appendix S2). The SMDs (95% CIs) for cTBS (R-DLPFC) + iTBS (L-DLPFC) and iTBS (L-DLPFC) were −0.947 ( − 1.502, −0.392) and −0.625 ( − 1.052, −0.198), respectively. Moreover, iTBS (L-DLPFC) had a higher remission rate compared the sham (RRs [95% CIs]) = 2.162 (1.012, 4.618), *k* = 15, Fig. 1, (Appendix S3). No significant differences were found for the all-cause discontinuation rate (*k* = 17, Fig. 1, Appendix S4), incidence of switch to mania (*k* = 7, Appendix S5), and incidence of headache/discomfort at treatment site (*k* = 10, Appendix S6) between TBS protocols included in each outcome and sham. Global heterogeneity was high in terms of depression symptom improvement and remission rate and low for all-cause discontinuation rate, incidence of switch to mania, and incidence of headache/discomfort at treatment site (Appendices S2–S6). Considerable local heterogeneity was noted for the depression symptom improvement and remission rate in a few specific comparisons (Appendix S2 and S3). Although we failed to evaluate global inconsistency for remission rate and switch to mania owing to the insufficient number of treatment loops, no significant global inconsistency for other outcomes was observed (Appendices S2–S6). Although we did not conduct the SIDE test for the remission rate and incidence of switch to mania due to the insufficiency of the number of treatment loops, we did not find significant local inconsistency for all comparisons in the depression symptom improvement, all-cause discontinuation rate, and incidence of headache/discomfort at treatment site (Appendices S2–S6).

In the majority of the comparisons, the within-study bias was assessed as “some concerns.” Moreover, because funnel plots with <10 studies were not meaningful [25], all comparisons other than iTBS (L-DLPFC) vs. sham for publication bias were evaluated as “some concerns.” Moreover, if the comparison only had an indirect evidence, the comparison was downgraded to one level. Consequently, confidence in the evidence was generally assessed as low or very low.

### Pairwise and single-group summary meta-analysis results

While iTBS (L-DLPFC) was superior to sham regarding the response rate (RR [95% CI] = 2.290 [1.437, 3.649]), no significant difference was found for the outcome between cTBS (R-DLPFC) + iTBS (L-DLPFC) and sham (RR [95% CI]) = 1.745 [0.926, 3.286], (Appendix S1). The exact response rate (95% CI) in cTBS (R-DLPFC) + iTBS (L-DLPFC) and sham was 43.42% (28.04, 60.18) and 25.80% (17.26, 36.68), respectively. The exact response rates (95% CI) for iTBS (L-DLPFC) and sham were 44.95% (31.82, 58.83) and 17.85% (10.58, 28.51), respectively.

The meta-regression analysis for cTBS (R-DLPFC) + iTBS (L-DLPFC) demonstrated that studies including individuals with TRD were associated with a larger effect size for the response rate than studies on individuals with no TRD (Appendix S1). Although in the sham group, the factor was not associated with the exact response rate, studies including individuals with TRD were associated with a more optimal response rate in the cTBS (R-DLPFC) + iTBS (L-DLPFC) group than studies including individuals with no TRD (Appendix S1). The meta-regression analysis for cTBS (R-DLPFC) + iTBS (L-DLPFC) revealed that studies with fewer pulses during a trial were associated with larger effect size for the response rate than studies with more pulses during a trial (Appendix S1). Although the number of pulses during a trial were not correlated with an exact response rate in the cTBS (R-DLPFC) + iTBS (L-DLPFC) group, studies with fewer pulses during a trial were associated with a lower exact response rate in the sham group compared with studies with more pulses during a trial (Appendix S1).

The meta-regression analysis for iTBS (L-DLPFC) showed that studies with more pulses during a session were associated with larger effect size for the response rate compared to studies with fewer pulses during a session (Appendix S1). Although the number of pulses during a session was not associated with exact response rate in the iTBS (L-DLPFC) group, studies with more pulses during a session were associated with a lower exact response rate in the sham group compared to studies with fewer pulses during a session (Appendix S1). Similar results were demonstrated in a subgroup including studies with 1000 and more pulses during a session, but not in another subgroup including studies with <1000 pulses (Appendix S1).

The sensitivity analysis for cTBS (R-DLPFC) + iTBS (L-DLPFC) excluding one RCT including individuals with mixed episode showed that cTBS (R-DLPFC) + iTBS (L-DLPFC) had a higher response rate compared to the sham (RR [95% CI] = 2.435 [1.537, 3.859], I^2^ = 0.00%). The sensitivity analysis for iTBS (L-DLPFC) excluding the SNT study also revealed that iTBS (L-DLPFC) had a higher response rate compared to the sham (RR [95% CI] = 2.111 [1.316, 3.386], I^2^ = 46.76%).

## Discussion

**Fig. 2:**
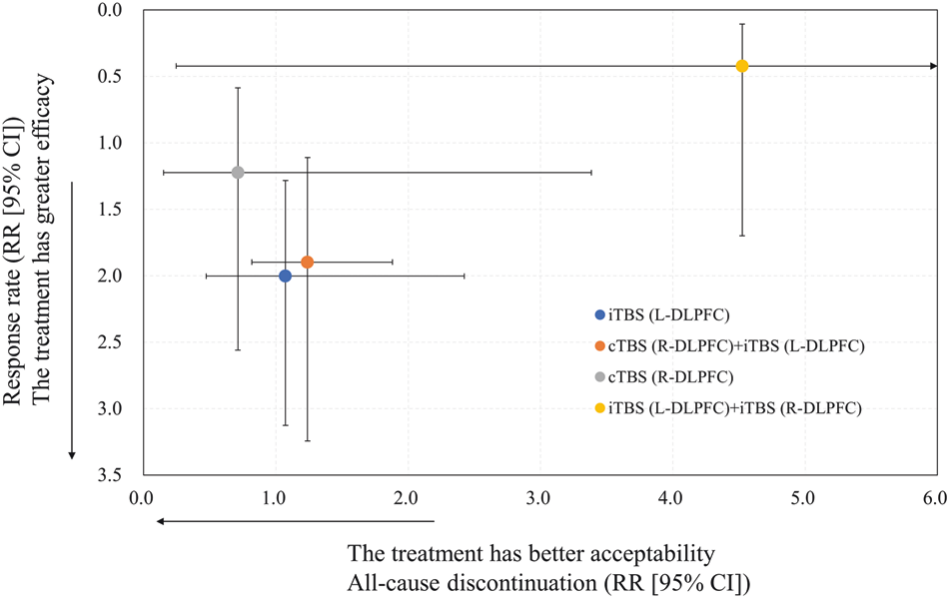
Two-dimensional Graph Regarding Efficacy and Acceptability. 95% CI: 95% confidence interval; cTBS: continuous theta burst stimulation; iTBS: intermittent theta burst stimulation; L (or R-) DLPFC: left (or right-) dorsolateral prefrontal cortex; RR: risk ratio.

This is the first systematic review and network meta-analysis comparing efficacy, acceptability, and safety of various TBS treatment protocols for individuals with depression. Our results suggest that iTBS (L-DLPFC) had a favorable risk-benefit balance for the treatment of depression because iTBS (L-DLPFC) had a high efficacy and better acceptability and safety profiles for individuals with depression. In Fig. 2, efficacy and acceptability are illustrated as a two-dimensional graph. We deem that cTBS (R-DLPFC) + iTBS (L-DLPFC) also improves symptoms in individuals with depression without mixed episodes, while iTBS (L-DLPFC) alone is a more straightforward choice and has become the most prominent. Furthermore, for individuals with depression, cTBS (R-DLPFC) alone had no therapeutic efficacy. Thus, cTBS (R-DLPFC) may not be suited for individuals with depression. However, as the number of participants and studies included in our meta-analysis was small, larger studies are warranted to generate a robust evidence.

One of our hypotheses was that the number of pulses administered is related to a greater antidepressant effect. This is supported by the fact that the SNT protocol showed the largest effect size for the primary outcome among all iTBS protocols targeting the L-DLPFC [11]. However, associations between the magnitude of the effect size for the response rate and number of pulses during the trial were not found, not only in cTBS (R-DLPFC) + iTBS (L-DLPFC) but also in iTBS (L-DLPFC). Studies with fewer pulses during a trial were associated with larger effect size for the response rate than studies with more pulses during a trial for cTBS (R-DLPFC) + iTBS (L-DLPFC), and the factor also only influenced the sham response (less pulses during a trial were associated with a lower response rate). For the iTBS (L-DLPFC) protocol, studies with a higher number of pulses during a session was associated with a larger effect size than studies with a fewer number of pulses during a session, and the factor also influenced the sham response (more pulses during a session were associated with a lower response rate). Furthermore, other factors related to the number of pulses were not associated with the effect size magnitude. Thus, our study results failed to elucidate the association between the number of pulses and the magnitude of antidepressant effect of TBS treatment. However, as SNT utilized a unique treatment protocol not used in other studies as mentioned above, differences in procedures (including targeting) between SNT and other studies could influence the magnitude of response.

For the accelerated iTBS protocol, in both active and sham iTBS groups, positive correlations were found between intersession interval and the exact response rate. However, because the coefficients of both treatments were similar, the intersession interval was not associated with the effect size of the primary outcome.

For iTBS (L-DLPFC), no significant difference in the effect size for the primary outcome between the MDD and BDep were found. Our meta-analysis revealed that rTMS is effective for BDep treatment [47]. Thus, iTBS (L-DLPFC) was considered as one of the treatments for MDD and BDep.

Although most studies included in our systematic review reported a high safety profile, the incidence of safety outcomes other than switch to mania and headache/discomfort at the treatment site was not reported. Although TBS protocols were considered as well-tolerated, because rTMS is known to rarely induce convulsions [48], clinicians must monitor individuals with depression.

Our network meta-analysis demonstrated that iTBS (occipital lobe) had a higher response rate compared the sham. However, no significant differences in the improvement of depression symptoms and remission rate between iTBS (occipital lobe) and sham were observed. Importantly, these results were just based on one small study [34]. Moreover, this study did not provide any available data on acceptability. Hence, larger trials might be needed to explore the efficacy and safety of iTBS (occipital lobe).

Our study had some limitations. First, the number of participants and the studies included in our meta-analysis were small compared with the meta-analysis for the pharmacological treatment [49–54]. Second, the study participants included in the meta-analysis took numerous drugs. For example, benzodiazepine use might impede the rTMS response, while a psychostimulant use might be associated with a greater response to rTMS [55, 56]. Third, we used the efficacy data from the day closest to the TMS treatment completion. If the antidepressant effect in the TBS treatment group persists and the antidepressant effect in the sham group is attenuated, the effect size could be greater when observed for over a prolonged period. Consequently, larger-scale, long-term studies of the TBS protocols are warranted to evaluate the longevity of its effects (e.g., through continuation study). Furthermore, our study did not address several considerations for informed choices in daily clinical practice, such as the integration with pharmacotherapy, other nonpharmacological interventions, and an analysis of cost-effectiveness.

In conclusion, cTBS (R-DLPFC) + iTBS (L-DLPFC) and iTBS (L-DLPFC) demonstrate a favorable risk-benefit balance for the treatment of depression.

## Supplementary information

Supplementary material

## Data Availability

Data used for the current study were reported in articles as cited in this paper.
