# Supplementary material for: Theta burst stimulation for depression: a systematic review and network and pairwise meta-analysis
Source: Mol Psychiatry. 2024 Jun 6;29(12):3893–9. doi: 10.1038/s41380-024-02630-5 (PMC11609094; doi:10.1038/s41380-024-02630-5)
Supplement: Supplementary file 1 — Supplementary material [file 41380_2024_2630_MOESM1_ESM.pdf]

Figure S1: PRISMA flow diagram

We searched the PubMed, the Cochrane Library, and Embase databases for studies published before August 4, 2023, without language restriction. The search terms for PubMed and the Cochrane Library included (depressi*) AND (theta burst stimulation OR TBS) AND (random*). The search terms for Embase included ('depression'/exp OR depression) AND ('randomized controlled trial'/exp OR 'randomized controlled trial') AND ('theta burst stimulation'/exp OR 'theta burst stimulation'). Additionally, reference lists of the included articles were manually searched for additional relevant published and unpublished research, including conference abstracts. We also searched clinical trial registries (ClinicalTrials.gov [http://clinicaltrials.gov/] and the World Health Organization International Clinical Trials Registry Platform [http://www.who.int/ictrp/search/en/]) to ensure the RCTs were comprehensive and to minimize the effect of publication bias. Any discrepancies in the selected articles were resolved by consensus of the authors. If multiple papers or academic conference abstracts were reported despite the same research, the literature was screened by confirming the clinical trial registration number and/or reference to past review articles.

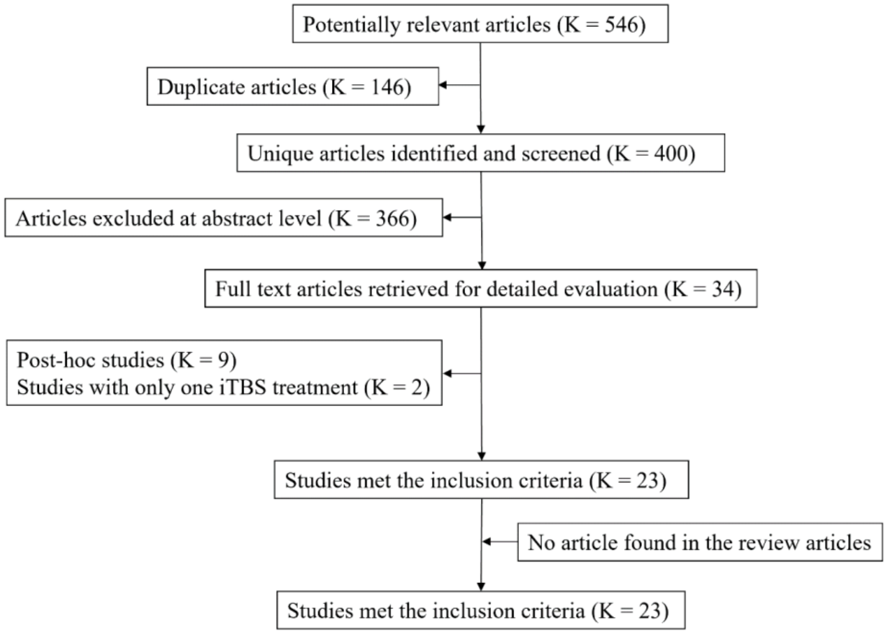

<u>Review articles that we read for the literature search</u>

1. Cai DB, Qin ZJ, Lan XJ, Liu QM, Qin XD, Wang JJ et al. Accelerated intermittent theta burst stimulation for major depressive disorder or bipolar depression: A systematic review and meta-analysis. Asian J Psychiatr 2023; 85: 103618.
2. Chu HT, Cheng CM, Liang CS, Chang WH, Juan CH, Huang YZ et al. Efficacy and tolerability of theta-burst stimulation for major depression: A systematic review and meta-analysis. Prog Neuropsychopharmacol Biol Psychiatry 2021; 106: 110168.
3. Hyde J, Carr H, Kelley N, Seneviratne R, Reed C, Parlatini V et al. Efficacy of neurostimulation across mental disorders: systematic review and meta-analysis of 208 randomized controlled trials. Mol Psychiatry 2022; 27(6): 2709-2719.
4. Konstantinou G, Hui J, Ortiz A, Kaster TS, Downar J, Blumberger DM et al. Repetitive transcranial magnetic stimulation (rTMS) in bipolar disorder: A systematic review. Bipolar Disord 2022; 24(1): 10-26.
5. Li H, Cui L, Li J, Liu Y, Chen Y. Comparative efficacy and acceptability of neuromodulation procedures in the treatment of treatment-resistant depression: a network meta-analysis of randomized controlled trials. J Affect Disord 2021; 287: 115-124.
6. McGirr A, Berlim MT. Clinical Usefulness of Therapeutic Neuromodulation for Major Depression: A Systematic Meta-Review of Recent Meta-Analyses. Psychiatr Clin North Am 2018; 41(3): 485-503.
7. McGirr A, Karmani S, Arsappa R, Berlim MT, Thirthalli J, Muralidharan K et al. Clinical efficacy and safety of repetitive transcranial magnetic stimulation in acute bipolar depression. World Psychiatry 2016; 15(1): 85-86.
8. Mutz J. Brain stimulation treatment for bipolar disorder. Bipolar Disord 2023; 25(1): 9-24.
9. Mutz J, Edgcumbe DR, Brunoni AR, Fu CHY. Efficacy and acceptability of non-invasive brain stimulation for the treatment of adult unipolar and bipolar depression: A systematic review and meta-analysis of randomised sham-controlled trials. Neurosci Biobehav Rev 2018; 92: 291-303.
10. Mutz J, Vipulananthan V, Carter B, Hurlemann R, Fu CHY, Young AH. Comparative efficacy and acceptability of non-surgical brain stimulation for the acute treatment of major depressive episodes in adults: systematic review and network meta-analysis. BMJ 2019; 364: l1079.
11. Rosson S, de Filippis R, Croatto G, Collantoni E, Pallottino S, Guinart D et al. Brain stimulation and other biological non-pharmacological interventions in mental disorders: An umbrella review. Neurosci Biobehav Rev 2022; 139: 104743.
12. Sonmez AI, Camsari DD, Nandakumar AL, Voort JLV, Kung S, Lewis CP et al. Accelerated TMS for Depression: A systematic review and meta-analysis. Psychiatry Res 2019; 273: 770-781.
13. Voigt JD, Leuchter AF, Carpenter LL. Theta burst stimulation for the acute treatment of major depressive disorder: A systematic review and meta-analysis. Transl Psychiatry 2021; 11(1): 330.

Table S1. Characteristics of the randomized, controlled trials in our systematic review

Study name	Design	Sponsorship	Country	Diagnosis	Minimum of depressive symptoms at baseline	Total n	Depressive symptoms score at baseline (mean±SD)	%Female	Age (mean±SD)	Treatment arms	Coil	Device	Locating	TBS treatment d	MT (%)	Frequency (Hz)	Total pulses/session	Total session/d	Total session	Total pulses	Intersession interval	Concomitant drug (%)
Beynel 2014	DBRSCT	Academia	France	TRBDep (100%, DSM-IV-TR)	MADRS>20	12	MADRS: 30.67±5.61	50	51.6±11.7	iTBS (L-DLPFC)	Figure-of-eight coil	MagProX100	MRI	15 d	80 (RMT)	50	990	2	30	29700	3 h	MS (NR), CY (NR), HY (NR)
										Sham coil												
Bulteau 2019	DBRSCT	Academia	France	TRBDep (100%, DSM-IV-TR)	MADRS>20	26	MADRS: 29.03±5.61	42.31	52.92±11.52	iTBS (L-DLPFC)	Figure-of-eight coil	MagProX100	MRI	15 d	80 (RMT)	50	990	2	30	29700	3 h	MS (100), AD (0), BE (0)
										Sham coil												
Cheng 2022	DBRSCT	Academia	Taiwan	TRMDD (100%, DSM-IV, MINI)	CGI-S>=4 and HDRS17>=18	34	HDRS17: 24.52±5.44	58.82	46.32±15.94	BTBS (600 pulses iTBS, L- and R-DMPFC)	Figure-of-eight coil	Magstim Rapid2	MRI	15 d	80 (AMT)	50	600(L)/600(R)	2	30	18000 (L)/18000(R)	15 min	No AD (100)
										BTBS (1800 pulses iTBS, L- and R-DMPFC)			MRI	15 d	80 (AMT)	50	1800(L)/1800(R)	2	30	54000(L)/54000(R)	15 min	
										Sham coil												
Chistyakov 2010	ORCT	Academia	Israel	TRMDD (NR)+TRBDep (NR) (DSM-IV)	No	13	HDRS: 27.35±4.97	84.62	54.65±14.97	iTBS (L-DLPFC)	Figure-of-eight coil	Magstim Super Rapid2	5 cm rule	10 d	90 (AMT)	50	600	2	20	12000	30 min	AD (NR), MS (NR)
										cTBS (R-DLPFC)	Figure-of-eight coil	Magstim Super Rapid2			90 (AMT)	50	600	2	20	12000	30 min	
Chistyakov 2015	DBRSCT	Academia	Israel	TRMDD (65.52%)+TRBDep (DSM-IV)	No	29	HDRS21: 25.78±3.65	62.07	51.83±14.19	cTBS (R-DLPFC)	Figure-of-eight coil	Magstim Super Rapid2	5 cm rule	10 d	100 (AMT)	50	900	4	40	36000	15 min	No med (10.34), AD (79.3), MS (48.3), AP (48.3)
										Sham coil												
Chou 2020	DBRSCT	Academia	Taiwan	TRMDD (100%, DSM-IV)	HDRS21>=18	60	HDRS21:24.55±4.60	60.38	42.96±14.05	BTBS (cTBS: R-DLPFC and iTBS: L-DLPFC)	Figure-of-eight coil	MAGSTIM 200	5 cm rule	10 d	80 (RMT)	50	600(L)/600(R)	1	10	6000(L)/6000(R)		No med (100)
										Sham coil												
Cole 2022	DBRSCT	Academia	USA	TRMDD (100%, DSM-5)	HDRS17 and MADRS>=20	29	MADRS: 33.07±5.44	34.48	50.55±15.32	iTBS (L-DLPFC)	Figure-of-eight coil	MagPro X100	MRI	5 d	90 (RMT)	50	1800	10	50	90000	50 min	AD (NR), BE (44.83), AC (NR), LI (13.79), AP (13.79) or No med (17.24)

										Sham coil												
Cristancho 2023	DBRSCT	Academia	USA	MDD and TRMDD (64.71%) with EFDs (DSM-5)	MADRS>=15	17	MADRS: 22.24±4.80	52.9	66.53±4.87	BTBS (iTBS: L- and R-DLPFC)	Figure-of-eight coil	Magpro R30	MRI	30 d	120 (RMT)	50	600(L)/600(R)	1	30	18000 (L)/18000 (R)		AD (76.47), BE (NR)
										Sham coil												
Duprat 2016	CODBRSCT	Academia	Belgium	TRMDD (100%, DSM-IV-TR or ICD-10, MINI)	HDRS17>=14	50	HDRS17: 21.34±5.26	70.21	41.72±11.80	iTBS (L-DLPFC)	Figure-of-eight coil	Magstim Rapid2 Plus1	MRI	4 d	110 (RMT)	50	1620	5	20	32400	15 min	No AD (100), BE (31.91)
										Sham coil												
Guan 2021	SBRSCT	Academia	China	MDD (100%, DSM-5)	HDRS17>=18	51	HDRS17:21.92±3.84	58.82	30.12±7.41	iTBS (Occipital lobe)	Figure-of-eight coil	MagPro R30	Oz point in 10/20 EEG System	20 d	120 (RMT)	50	600	2	20	12000		ESC (100)
										Sham (the rTMS coil was perpendicular to the occipital region)												
Holczer 2021	DBRSCT	Academia	Hungary	MDD (100%, DSM-IV, SCID)	No	25	HAMD21:17.25 ± 5.43	75.00	50.27±13.24	BTBS (cTBS: R-DLPFC and iTBS: L-DLPFC)	Figure-of-eight coil	Magstim Rapid2	MRI	10 d	30 (RMT)	50	600(L)/600(R)	1	10	6000(L)/6000(R)		AD (85.0), BE (70.0)
										Sham (90° coil angulation)												
Li 2014	DBRSCT	Academia	Taiwan	TRMDD (100%, DSM-IV, MINI)	CGI-S>=4, HDRS17>=18	60	HDRS17:24.15±4.48	66.67	45.25	cTBS (R-DLPFC)	Figure-of-eight coil	Magstim Rapid2	MRI	10 d	80 (AMT)	50	1800	1	10	18000		AD (NR), AP (41.67), BE (71.67), No med (25)
										iTBS (L-DLPFC)					80 (AMT)	50	1800	1	10	18000		
										BTBS (cTBS: R-DLPFC and iTBS: L-DLPFC)					80 (AMT)	50	1800(L)/1800(R)	1	10	18000(L)/18000(R)		
										Sham (90° coil angulation)								1	10			
Li 2020	DBRSCT	Academia	Taiwan	TRMDD (100%, DSM-IV)	CGI-S>=4, HDRS17>=18	70	HDRS17:22.8±3.49	67.14	47.1±13.23	iTBS (L-DLPFC)	Figure-of-eight coil	Magstim Rapid2	MRI or 5 cm rule	10 d	80 (AMT)	50	1800	1	10	18000		No AD (100)
										Sham coil												
Li 2023	DBRSCT	Academia	Taiwan	TRMDD (100%, DSM-IV, MINI)	CGI-S>=4, HDRS17>=18	48	HDRS17:24.3±1.11	64.58	37.00±2.88	iTBS (L-DLPFC)	Figure-of-eight coil	Magstim Rapid2	MRI	10 d	80 (AMT)	50	1800	2	20	36000	0	No AD (100)
										Sham coil								2	20			

Mallik 2023	SBRSCT	Academia	India	BDep (100%, ICD-10)	HDRS>18	19	HDRS: 27.05±8.87	47.37	39.48±12.43	cTBS (R-DLPFC)	Figure-of-eight coil	MagPro R30	F4 in 10/20 EEG System	5 d	80 (RMT)	50	600	3	15	9000	30 min	No med (21.05), AD (10.53), AD+MS (42.11), MS (26.32)
										Sham coil												
McGirr 2021	DBRSCT	Academia	Canada	TRBDep (100%, DSM-5)	HDRS17>=18	37	MADRS:32.97±4.19	62.16	43.86±13.87	iTBS (L-DLPFC)	Figure-of-eight coil	MagPro X100	MRI or BeamF3	20 d	120 (RMT)	50	600	1	20	12000		MS (43.24), SGA (8.11), MS+SGA (48.65)
										Sham coil												
Murgaš 2023	DBRSCT	Academia	Austria	TRMDD (100%, DSM-5, SCID)	HDRS17>=18	11	HDRS: 19.45±3.59	72.73	37.09±7.82	BTBS (cTBS: R-DLPFC and iTBS: L-DLPFC)	Figure-of-eight coil	MagPro X100	MRI	15 d	120 (RMT)	50	600(L)/600(R)	2	30	18000(L)/18000(R)		AD (100), AP (36.36), MS (36.36)
										Sham (90° coil angulation)												
Plewnia 2014	DBRSCT	Academia	Germany	MDD and TRMDD (68.75%, DSM-IV)	No	32	MADRS:26.70±6.99	62.50	47.95±13.23	BTBS (cTBS: R-DLPFC and iTBS: L-DLPFC)	Figure-of-eight coil	Magstim Super Rapid	F3 and F4 in 10/20 EEG System	30 d	80 (RMT)	50	600(L)/600(R)	1	30	18000(L)/18000(R)		AD (NR), QUE (3.13), LIT (3.13)
										Sham (45° coil angulation)												
Prasser 2015	DBRSCT	Academia	Germany	MDD (100%, ICD10)	HDRS21>=18	38	HDRS21:26.44±6.03	51.35	45.63±11.79	BTBS (cTBS: R-DLPFC and iTBS: L-DLPFC)	Figure-of-eight coil	MagPro X100	Anterior to the motor hotspot (6 cm)	15 d	80 (RMT)	50	1200(L)/1200(R)	1	15	18000(L)/18000(R)		AD (NR), AP (54.05), LI (13.51), AC (16.22)
										Sham coil												
Tavares 2021	DBRSCT	Academia	Brazil	MDD (47.8%) and BD with mixed features (DSM-5, SCID)	MADRS>=18	100	MADRS:34.96±6.05	71.11	39.4±10.4	BTBS (cTBS: R-DLPFC and iTBS: L-DLPFC)	Figure-of-eight coil	MagPro X100	F3 and F4 in 10/20 EEG System	21 d	80 (RMT)	50	1800(L)/1800(R)	1	21	37800(L)/37800(R)		AD(NR), SGA (NR), LI (NR), AC (NR)
										Sham coil												
Wilkening 2022	COQBRSCT	Academia	Germany	MDD (100%, DSM-5, SCID)	No	92	MADRS:23.31±7.50	41.98	35.65±13.03	iTBS (L-DLPFC)	Figure-of-eight coil	MagVenture X100	MRI	5 d	110 (RMT)	50	1800	4	20	36000	>=20 min	No med (27.16), AD(NR), LI (2.47), AC (2.47), BE(3.70)
										Sham (180° coil angulation)												
Zavorotnyy 2020	DBRSCT	Academia	Germany	MDD (100%, DSM-IV, SCID)	No	57	HDRS21:16.55±5.38	52.73	43.00±12.68	iTBS (L-DLPFC)	Figure-of-eight coil	MagPro X100	F3 in 10/20 EEG System	20 d	90 (RMT)	50	600	2	40	24000	10 min	No med (3.64), AD(NR), LI (7.27), AC (0),

																						AP (7.27), BE (0)
										Sham coil												
Zhao 2023	SBRSCT	Academia	China	MDD (100%, DSM-IV-TR, SCID)	HDRS24>=20	50	HDRS24:27.17±6.30	82.22	17.20±2.25	iTBS (L-DLPFC)	Figure-of-eight coil	YRD CCY-I magnetic simulator	5 cm rule	10 d	80 (AMT)	50	1800	1	10	18000		AD (100)
										Sham coil												

AC: anticonvulsant, AD: antidepressant, AMT: active motor threshold, AP: antipsychotic, BD: bipolar disorder, BE: benzodiazepine(s), BTBS: bilateral theta burst stimulation, CGI-S: Clinical Global Impression-Severity score, CODBRSCT: crossover double-blind randomized sham-controlled trial, cTBS: continuous theta burst stimulation, CY: cyamemazine, DBRSCT: double-blind randomized sham-controlled trial, DSM(-TR): Diagnostic and Statistical Manual of Mental Disorders (-Text Revision), EEG: electroencephalography, ESC: escitalopram, HDRS: Hamilton Depression Rating Scale, HY: hydroxyzine, Hz: hertz, ICD: International Statistical Classification of Diseases and Related Health Problems, iTBS: intermittent theta burst stimulation, L-, R-DLPFC: left-, right- dorsolateral prefrontal cortex, L-, R-DMPFC: left-, right- dorsomedial prefrontal cortex, LI: lithium, MADRS: Montgomery Åsberg Depression Rating Scale, MINI: Mini-International Neuropsychiatric Interview, med: medication, MRI: magnetic resonance imaging, MS: mood stabilizer(s), MT: motor threshold, n: number of individuals, NR= not report, QUE: quetiapine, R-DLPFC: right-dorsolateral prefrontal cortex, RMT: resting motor threshold, SBRSCT: single-blind randomized sham-controlled trial, SD: standard deviation, SGA: second generation antipsychotic, TRBDep: treatment resistant bipolar depression, TRMDD: treatment resistant major depressive disorder

Table S2-1. PRISMA for Network Meta-Analyses Checklist.

Section/Topic	Item #	Checklist Item	Reported on Page #
**TITLE**			
Title	1	Identify the report as a systematic review *incorporating a network meta-analysis (or related form of meta-analysis).*	1
**ABSTRACT**			
Structured summary	2	Provide a structured summary including, as applicable:<br>**Background:** main objectives<br>**Methods:** data sources; study eligibility criteria, participants, and interventions; study appraisal; and *synthesis methods, such as network meta-analysis.*<br>**Results:** number of studies and participants identified; summary estimates with corresponding confidence/credible intervals; *treatment rankings may also be discussed. Authors may choose to summarize pairwise comparisons against a chosen treatment included in their analyses for brevity.*<br>**Discussion/Conclusions:** limitations; conclusions and implications of findings.<br>**Other:** primary source of funding; systematic review registration number with registry name.	3-
**INTRODUCTION**			
Rationale	3	Describe the rationale for the review in the context of what is already known, *including mention of why a network meta-analysis has been conducted.*	5-
Objectives	4	Provide an explicit statement of questions being addressed, with reference to participants, interventions, comparisons, outcomes, and study design (PICOS).	5-
**METHODS**			
Protocol and registration	5	Indicate whether a review protocol exists and if and where it can be accessed (e.g., Web address); and, if available, provide registration information, including registration number.	7-
Eligibility criteria	6	Specify study characteristics (e.g., PICOS, length of follow-up) and report characteristics (e.g., years considered, language, publication status) used as criteria for eligibility, giving rationale. *Clearly describe eligible treatments included in the treatment network, and note whether any have been clustered or merged into the same node (with justification).*	7-
Information sources	7	Describe all information sources (e.g., databases with dates of coverage, contact with study authors to identify additional studies) in the search and date last searched.	7-
Search	8	Present full electronic search strategy for at least one database, including any limits used, such that it could be repeated.	7-
Study selection	9	State the process for selecting studies (i.e., screening, eligibility, included in systematic review, and, if applicable, included in the meta-analysis).	7-
Data collection process	10	Describe method of data extraction from reports (e.g., piloted forms, independently, in duplicate) and any processes for obtaining and confirming data from investigators.	7-
Data items	11	List and define all variables for which data were sought (e.g., PICOS, funding sources) and any assumptions and simplifications made.	7-

**Geometry of the network**	**S1**	Describe methods used to explore the geometry of the treatment network under study and potential biases related to it. This should include how the evidence base has been graphically summarized for presentation, and what characteristics were compiled and used to describe the evidence base to readers.	7-
Risk of bias within individual studies	12	Describe methods used for assessing risk of bias of individual studies (including specification of whether this was done at the study or outcome level), and how this information is to be used in any data synthesis.	7-
Summary measures	13	State the principal summary measures (e.g., risk ratio, difference in means). *Also describe the use of additional summary measures assessed, such as treatment rankings and surface under the cumulative ranking curve (SUCRA)* values, as well as modified approaches used to present summary findings from meta-analyses.*	7-
Planned methods of analysis	14	Describe the methods of handling data and combining results of studies for each network meta-analysis. This should include, but not be limited to:<br>• *Handling of multi-arm trials;*<br>• *Selection of variance structure;*<br>• *Selection of prior distributions in Bayesian analyses; and*<br>• *Assessment of model fit.*	7-
**Assessment of Inconsistency**	**S2**	Describe the statistical methods used to evaluate the agreement of direct and indirect evidence in the treatment network(s) studied. Describe efforts taken to address its presence when found.	7-
Risk of bias across studies	15	Specify any assessment of risk of bias that may affect the cumulative evidence (e.g., publication bias, selective reporting within studies).	7-
Additional analyses	16	Describe methods of additional analyses if done, indicating which were pre-specified. This may include, but not be limited to, the following:<br>• Sensitivity or subgroup analyses;<br>• Meta-regression analyses;<br>• *Alternative formulations of the treatment network; and*<br>• *Use of alternative prior distributions for Bayesian analyses (if applicable).*	7-
**RESULTS†**			
Study selection	17	Give numbers of studies screened, assessed for eligibility, and included in the review, with reasons for exclusions at each stage, ideally with a flow diagram.	11-
**Presentation of network structure**	**S3**	Provide a network graph of the included studies to enable visualization of the geometry of the treatment network.	11-
**Summary of network geometry**	**S4**	Provide a brief overview of characteristics of the treatment network. This may include commentary on the abundance of trials and randomized patients for the different interventions and pairwise comparisons in the network, gaps of evidence in the treatment network, and potential biases reflected by the network structure.	11-
Study characteristics	18	For each study, present characteristics for which data were extracted (e.g., study size, PICOS, follow-up period) and provide the citations.	11-
Risk of bias within studies	19	Present data on risk of bias of each study and, if available, any outcome level assessment.	11-

	Results of individual studies	20	For all outcomes considered (benefits or harms), present, for each study: 1) simple summary data for each intervention group, and 2) effect estimates and confidence intervals. *Modified approaches may be needed to deal with information from larger networks.*	11-
	Synthesis of results	21	Present results of each meta-analysis done, including confidence/credible intervals. *In larger networks, authors may focus on comparisons versus a particular comparator (e.g. placebo or standard care), with full findings presented in an appendix. League tables and forest plots may be considered to summarize pairwise comparisons.* If additional summary measures were explored (such as treatment rankings), these should also be presented.	11-
	**Exploration for inconsistency**	**S5**	Describe results from investigations of inconsistency. This may include such information as measures of model fit to compare consistency and inconsistency models, *P* values from statistical tests, or summary of inconsistency estimates from different parts of the treatment network.	11-
	Risk of bias across studies	22	Present results of any assessment of risk of bias across studies for the evidence base being studied.	11-
	Results of additional analyses	23	Give results of additional analyses, if done (e.g., sensitivity or subgroup analyses, meta-regression analyses, *alternative network geometries studied, alternative choice of prior distributions for Bayesian analyses*, and so forth).	11-
**DISCUSSION**				
	Summary of evidence	24	Summarize the main findings, including the strength of evidence for each main outcome; consider their relevance to key groups (e.g., healthcare providers, users, and policy-makers).	15-
	Limitations	25	Discuss limitations at study and outcome level (e.g., risk of bias), and at review level (e.g., incomplete retrieval of identified research, reporting bias). *Comment on the validity of the assumptions, such as transitivity and consistency. Comment on any concerns regarding network geometry (e.g., avoidance of certain comparisons).*	15-
	Conclusions	26	Provide a general interpretation of the results in the context of other evidence, and implications for future research.	15-
**FUNDING**				19
	Funding	27	Describe sources of funding for the systematic review and other support (e.g., supply of data); role of funders for the systematic review. This should also include information regarding whether funding has been received from manufacturers of treatments in the network and/or whether some of the authors are content experts with professional conflicts of interest that could affect use of treatments in the network.	

Table S2-2. PRISMA for Pairwise Meta-Analyses Checklist.

Section and Topic	Item #	Checklist item	Location where item is reported
**TITLE**			
Title	1	Identify the report as a systematic review.	1
**ABSTRACT**			
Abstract	2	See the PRISMA 2020 for Abstracts checklist.	3
**INTRODUCTION**			
Rationale	3	Describe the rationale for the review in the context of existing knowledge.	5-
Objectives	4	Provide an explicit statement of the objective(s) or question(s) the review addresses.	5-
**METHODS**			
Eligibility criteria	5	Specify the inclusion and exclusion criteria for the review and how studies were grouped for the syntheses.	7-
Information sources	6	Specify all databases, registers, websites, organisations, reference lists and other sources searched or consulted to identify studies. Specify the date when each source was last searched or consulted.	7-
Search strategy	7	Present the full search strategies for all databases, registers and websites, including any filters and limits used.	7-
Selection process	8	Specify the methods used to decide whether a study met the inclusion criteria of the review, including how many reviewers screened each record and each report retrieved, whether they worked independently, and if applicable, details of automation tools used in the process.	7-
Data collection process	9	Specify the methods used to collect data from reports, including how many reviewers collected data from each report, whether they worked independently, any processes for obtaining or confirming data from study investigators, and if applicable, details of automation tools used in the process.	7-
Data items	10a	List and define all outcomes for which data were sought. Specify whether all results that were compatible with each outcome domain in each study were sought (e.g. for all measures, time points, analyses), and if not, the methods used to decide which results to collect.	7-
	10b	List and define all other variables for which data were sought (e.g. participant and intervention characteristics, funding sources). Describe any assumptions made about any missing or unclear information.	7-
Study risk of bias assessment	11	Specify the methods used to assess risk of bias in the included studies, including details of the tool(s) used, how many reviewers assessed each study and whether they worked independently, and if applicable, details of automation tools used in the process.	7-
Effect measures	12	Specify for each outcome the effect measure(s) (e.g. risk ratio, mean difference) used in the synthesis or presentation of results.	7-
Synthesis methods	13a	Describe the processes used to decide which studies were eligible for each synthesis (e.g. tabulating the study intervention characteristics and comparing against the planned groups for each synthesis (item #5)).	7-
	13b	Describe any methods required to prepare the data for presentation or synthesis, such as handling of missing summary statistics, or data conversions.	7-
	13c	Describe any methods used to tabulate or visually display results of individual studies and syntheses.	7-
	13d	Describe any methods used to synthesize results and provide a rationale for the choice(s). If meta-analysis was performed, describe the model(s), method(s) to identify the presence and extent of statistical heterogeneity, and software package(s) used.	7-
	13e	Describe any methods used to explore possible causes of heterogeneity among study results (e.g. subgroup analysis, meta-regression).	7-
	13f	Describe any sensitivity analyses conducted to assess robustness of the synthesized results.	7-
Reporting bias assessment	14	Describe any methods used to assess risk of bias due to missing results in a synthesis (arising from reporting biases).	7-

Section and Topic	Item #	Checklist item	Location where item is reported
Certainty assessment	15	Describe any methods used to assess certainty (or confidence) in the body of evidence for an outcome.	7-
**RESULTS**			
Study selection	16a	Describe the results of the search and selection process, from the number of records identified in the search to the number of studies included in the review, ideally using a flow diagram.	11-
	16b	Cite studies that might appear to meet the inclusion criteria, but which were excluded, and explain why they were excluded.	11-
Study characteristics	17	Cite each included study and present its characteristics.	11-
Risk of bias in studies	18	Present assessments of risk of bias for each included study.	11-
Results of individual studies	19	For all outcomes, present, for each study: (a) summary statistics for each group (where appropriate) and (b) an effect estimate and its precision (e.g. confidence/credible interval), ideally using structured tables or plots.	11-
Results of syntheses	20a	For each synthesis, briefly summarise the characteristics and risk of bias among contributing studies.	11-
	20b	Present results of all statistical syntheses conducted. If meta-analysis was done, present for each the summary estimate and its precision (e.g. confidence/credible interval) and measures of statistical heterogeneity. If comparing groups, describe the direction of the effect.	11-
	20c	Present results of all investigations of possible causes of heterogeneity among study results.	11-
	20d	Present results of all sensitivity analyses conducted to assess the robustness of the synthesized results.	11-
Reporting biases	21	Present assessments of risk of bias due to missing results (arising from reporting biases) for each synthesis assessed.	11-
Certainty of evidence	22	Present assessments of certainty (or confidence) in the body of evidence for each outcome assessed.	11-
**DISCUSSION**			
Discussion	23a	Provide a general interpretation of the results in the context of other evidence.	15-
	23b	Discuss any limitations of the evidence included in the review.	15-
	23c	Discuss any limitations of the review processes used.	15-
	23d	Discuss implications of the results for practice, policy, and future research.	15-
**OTHER INFORMATION**			
Registration and protocol	24a	Provide registration information for the review, including register name and registration number, or state that the review was not registered.	7
	24b	Indicate where the review protocol can be accessed, or state that a protocol was not prepared.	7
	24c	Describe and explain any amendments to information provided at registration or in the protocol.	7
Support	25	Describe sources of financial or non-financial support for the review, and the role of the funders or sponsors in the review.	19
Competing interests	26	Declare any competing interests of review authors.	19
Availability of data, code and other materials	27	Report which of the following are publicly available and where they can be found: template data collection forms; data extracted from included studies; data used for all analyses; analytic code; any other materials used in the review.	19

From: Page MJ, McKenzie JE, Bossuyt PM, Boutron I, Hoffmann TC, Mulrow CD, et al. The PRISMA 2020 statement: an updated guideline for reporting systematic reviews. BMJ 2021;372:n71. doi: 10.1136/bmj.n71

For more information, visit: http://www.prisma-statement.org/

Table S3. Results of the original study and data synthesis of our meta-analysis

Study	The definition of response	Results of the study*	The definition of remission	Results of the study*	Depression scale	Results of the study*
Beynel 2014	MADRS>=50%	iTBS (L-DLPFC) = Sham	MADRS=<7	iTBS (L-DLPFC) vs Sham: NR	MADRS	iTBS (L-DLPFC) = Sham
Bulteau 2019	MADRS>=50%	iTBS (L-DLPFC) = Sham	MADRS=<7	iTBS L-DLPFC = Sham	MADRS	iTBS (L-DLPFC) = Sham
Cheng 2022	HDRS17>50%	NR	HDRS17=<7	NR	HDRS17	BTBS (iTBS: L- and R-DMPFC) = Sham
Chistyakov 2010	HDRS>=50%	iTBS (L-DLPFC) = cTBS (R-DLPFC)			HDRS	iTBS (L-DLPFC) = cTBS (R-DLPFC)
Chistyakov 2015	HDRS21>=50%	cTBS (R-DLPFC) = Sham			HDRS21	cTBS (R-DLPFC) = Sham
Chou 2020	HDRS21>=50%	BTBS (cTBS: R-DLPFC and iTBS: L-DLPFC) > Sham	HDRS21=<8	BTBS (iTBS: L-DLPFC and cTBS: R-DLPFC) > Sham	HDRS21	BTBS (cTBS: R-DLPFC and iTBS: L-DLPFC) > Sham
Cole 2022	MADRS>=50%	iTBS (L-DLPFC) > Sham	MADRS=<10	iTBS (L-DLPFC) > Sham	MADRS	iTBS (L-DLPFC) > Sham
Cristancho 2023	MADRS>=50%	Sham > BTBS (iTBS: L- and R-DLPFC)			MADRS	BTBS (iTBS: L- and R-DLPFC) = Sham
Duprat 2016	HDRS17>=50%	iTBS (L-DLPFC) = Sham	HDRS17=<7	iTBS (L-DLPFC) vs Sham: NR	HDRS17	iTBS (L-DLPFC) = Sham
Guan 2021	HDRS17>=50%	NR	HDRS17=<7	NR	HDRS17	iTBS (occipital lobe) = Sham
Holczer 2021					HDRS21	BTBS (cTBS: R-DLPFC and iTBS: L-DLPFC) > Sham
Li 2014	HDRS17>=50%	cTBS (R-DLPFC) = Sham, iTBS (L-DLPFC) > Sham, BTBS (cTBS: R-DLPFC and iTBS: L-DLPFC) > Sham			HDRS17	cTBS (R-DLPFC) = Sham, iTBS (L-DLPFC) > Sham, BTBS (cTBS: R-DLPFC and iTBS: L-DLPFC) > Sham
Li 2020	HDRS17>=50%	NR	HDRS17=<7	NR	HDRS17	iTBS (L-DLPFC) > Sham
Li 2023	HDRS17>=50%	iTBS (L-DLPFC) > Sham	HDRS17=<7	iTBS (L-DLPFC) = Sham	HDRS17	iTBS (L-DLPFC) = Sham
Mallik 2023	HDRS>=50%	NR	HRSD=<7	NR	HDRS	cTBS (R-DLPFC) = Sham
McGirr 2021	MADRS>=50%	iTBS (L-DLPFC) = Sham	MADRS<=12	iTBS (L-DLPFC) = Sham	MADRS	iTBS (L-DLPFC) = Sham
Murgaš 2023	HDRS>=50%	NR			HDRS17	NR
Plewnia 2014	MADRS>=50%	BTBS (cTBS: R-DLPFC and iTBS: L-DLPFC) > Sham	MADRS=<7	BTBS (cTBS: R-DLPFC and iTBS: L-DLPFC) = Sham	MADRS	NR: BTBS (cTBS: R-DLPFC and iTBS: L-DLPFC) vs Sham
Prasser 2015	HDRS21>=50%	BTBS (cTBS: R-DLPFC and iTBS: L-DLPFC) = Sham	HDRS21=<10	NR	HDRS21	BTBS (cTBS: R-DLPFC and iTBS: L-DLPFC) = Sham
Tavares 2021	MADRS>=50%	BTBS (cTBS: R-DLPFC and iTBS: L-DLPFC) = Sham	MADRS=<10	BTBS (cTBS: R-DLPFC and iTBS: L-DLPFC) = Sham	MADRS	BTBS (cTBS: R-DLPFC and iTBS: L-DLPFC) = Sham
Wilkening 2022**	MADRS>=50%	iTBS (L-DLPFC) = Sham	MADRS<=7	iTBS (L-DLPFC) = Sham	MADRS	iTBS (L-DLPFC) = Sham
Zavorotnyy 2020	HDRS21>50%	iTBS (L-DLPFC) = Sham	HDRS21=<6	iTBS (L-DLPFC) = Sham	HDRS21	iTBS (L-DLPFC) = Sham

Zhao 2023	HDRS24>=50%	NR	HDRS24=<7	NR	HDRS24	iTBS (L-DLPFC) > Sham

We used efficacy data from the day closest to the completion of the TMS treatment.

*A = B: A was similar to B, A > B: A was superior to B.

**The study demonstrated that iTBS (L-DLPFC) was superior to sham regarding some efficacy outcomes at 1 week after the end of treatment. Please see the detailed results in the following article: Suicidality and relief of depressive symptoms with intermittent theta burst stimulation in a sham-controlled randomized clinical trial. Wilkening J, Witteler F, Goya-Maldonado R. Acta Psychiatr Scand. 2022 Dec;146(6):540-556.

BTBS: bilateral theta burst stimulation, cTBS: continuous theta burst stimulation, HDRS: Hamilton Depression Rating Scale, L-, R-DLPFC: left-, right- dorsolateral prefrontal cortex, L-, R-DMPFC: left-, right- dorsomedial prefrontal cortex, iTBS: intermittent theta burst stimulation, MADRS: Montgomery Åsberg Depression Rating Scale, NR: not report the statistical result

Table S4. Transitivity assessment.

	Boxplot	Kruskal–Wallis equality of populations rank test for continuous variables or the Pearson chi-squared test for binary and categorical variables (or the Fisher exact test whether >20% of cells had an expected frequency below 5).
Mean age (k = 23)	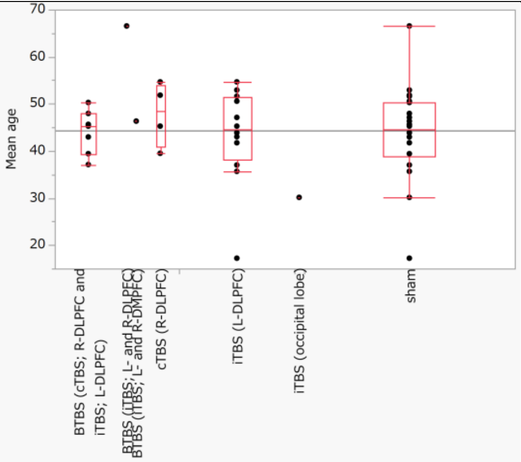	Chi-squared with ties = 6.22 (df = 6), p = 0.399
Proportion of females (k = 23)	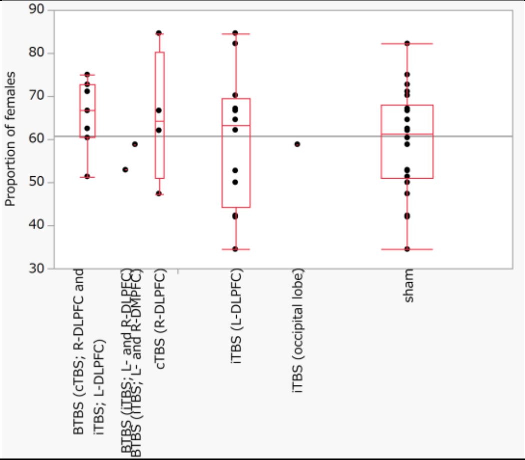	Chi-squared with ties = 2.79 (df = 6), p = 0.835

Total number of participants (k = 23)	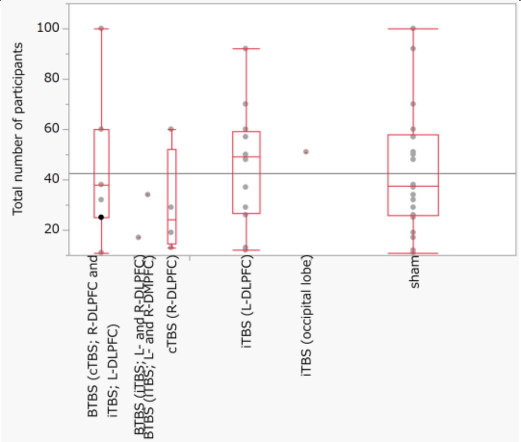	Chi-squared with ties = 3.19 (df = 6), p = 0.785
Minimum depressive symptoms at baseline (k = 23)	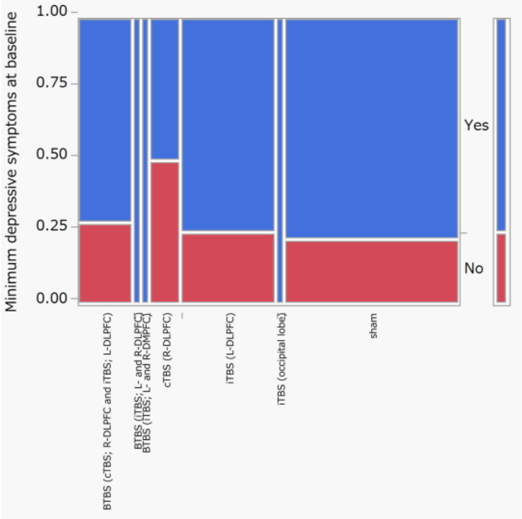	Chi-squared with ties = 2.99 (df = 6), p = 0.811

<table>
<tr>
<td>Diagnosis (k = 23)</td>
<td>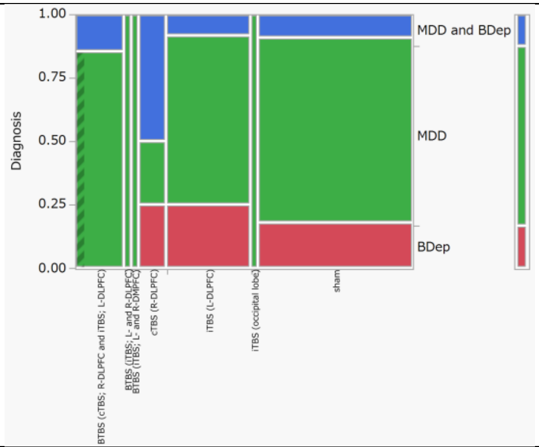
</td>
<td>Chi-squared with ties = 9.82 (df = 12), p = 0.632</td>
</tr>
<tr>
<td>Publication year (k = 23)</td>
<td>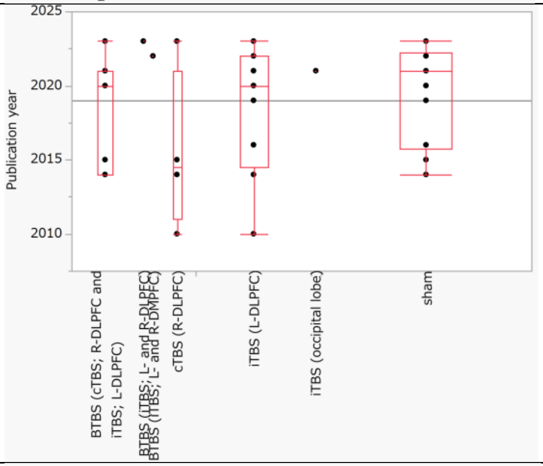
</td>
<td>Chi-squared with ties = 5.06 (df = 6), p = 0.536</td>
</tr>
<tr>
<td>Overall risk of bias (k = 23)</td>
<td>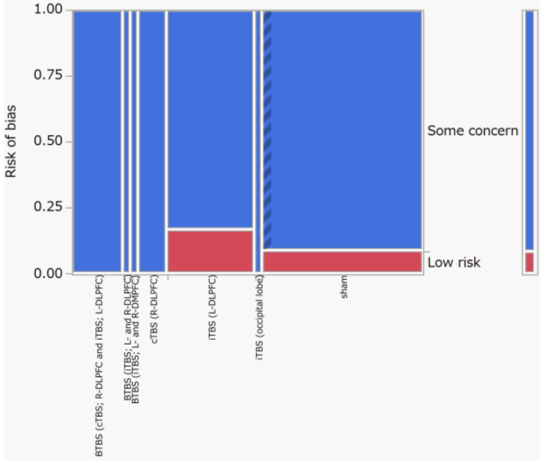
</td>
<td>Chi-squared with ties = 3.32 (df = 6), p = 0.768</td>
</tr>
</table>

Locating method (k = 23)	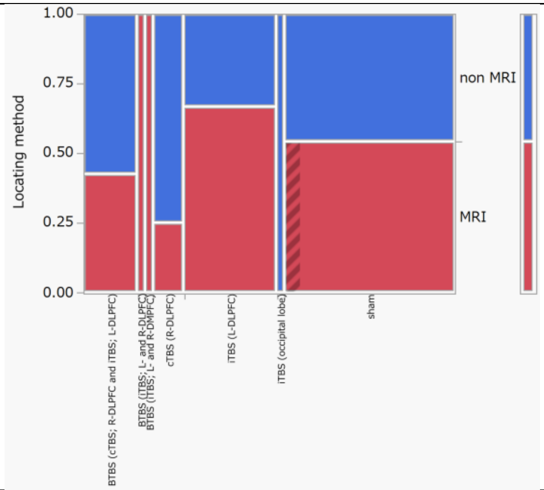	Chi-squared with ties = 6.56 (df = 6), p = 0.364
Total number of sessions during the study (k = 23)	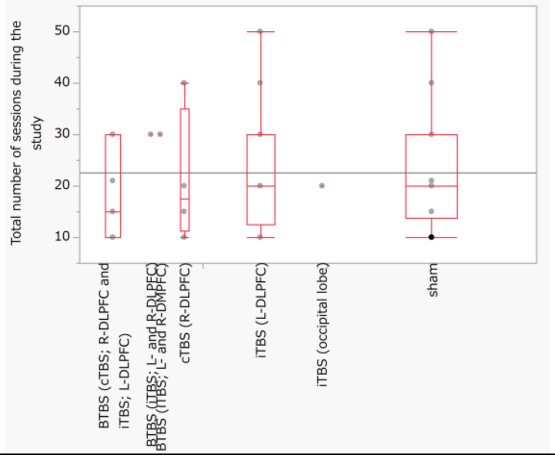	Chi-squared with ties = 2.74 (df = 6), p = 0.841

Table S5: Risk of bias summary

	Randomization process	Deviation from intended intervention	Missing outcome data	Measurement of the outcome	Selection of the reported result	Overall risk of bias
Beynel 2014	Some concerns	Low risk	Low risk	Low risk	Some concerns	Some concerns
Bulteau 2019	Some concerns	Low risk	Low risk	Low risk	Some concerns	Some concerns
Cheng 2022	Some concerns	Low risk	Low risk	Low risk	Low risk	Some concerns
Chistyakov 2010	Some concerns	Some concerns	Low risk	Some concerns	Some concerns	Some concerns
Chistyakov 2015	Some concerns	Some concerns	Low risk	Low risk	Low risk	Some concerns
Chou 2020	Some concerns	Some concerns	Some concerns	Low risk	Low risk	Some concerns
Cole 2022	Low risk	Low risk	Low risk	Low risk	Low risk	Low risk
Cristancho 2023	Some concerns	Low risk	Low risk	Low risk	Low risk	Some concerns
Duprat 2016	Some concerns	Some concerns	Low risk	Low risk	Low risk	Some concerns
Guan 2021	Low risk	Low risk	Low risk	Some concerns	Some concerns	Some concerns
Holczer 2021	Some concerns	Low risk	Low risk	Some concerns	Low risk	Some concerns
Li 2014	Some concerns	Low risk	Low risk	Low risk	Some concerns	Some concerns
Li 2020	Some concerns	Low risk	Low risk	Low risk	Low risk	Some concerns
Li 2023	Some concerns	Low risk	Low risk	Low risk	Low risk	Some concerns
Mallik 2023	Low risk	Some concerns	Low risk	Low risk	Low risk	Some concerns
McGirr 2021	Low risk	Some concerns	Low risk	Low risk	Low risk	Low risk
Murgaš 2023	Some concerns	Some concerns	Low risk	Low risk	Low risk	Some concerns
Plewnia 2014	Some concerns	Some concerns	Low risk	Low risk	Low risk	Some concerns
Prasser 2015	Some concerns	Some concerns	Low risk	Low risk	Low risk	Some concerns
Tavares 2021	Low risk	Low risk	Low risk	Low risk	Low risk	Some concerns
Wilkening 2022	Some concerns	Low risk	Low risk	Low risk	Some concerns	Some concerns
Zavorotnyy 2020	Some concerns	Some concerns	Some concerns	Low risk	Some concerns	Some concerns
Zhao 2023	Some concerns	Some concerns	Some concerns	Some concerns	Some concerns	Some concerns

Version 2 of the Cochrane risk-of-bias tool for randomized trials (https://www.riskofbias.info/)

Appendix S1. Response rate

Network meta-analysis

23 studies, 964 participants

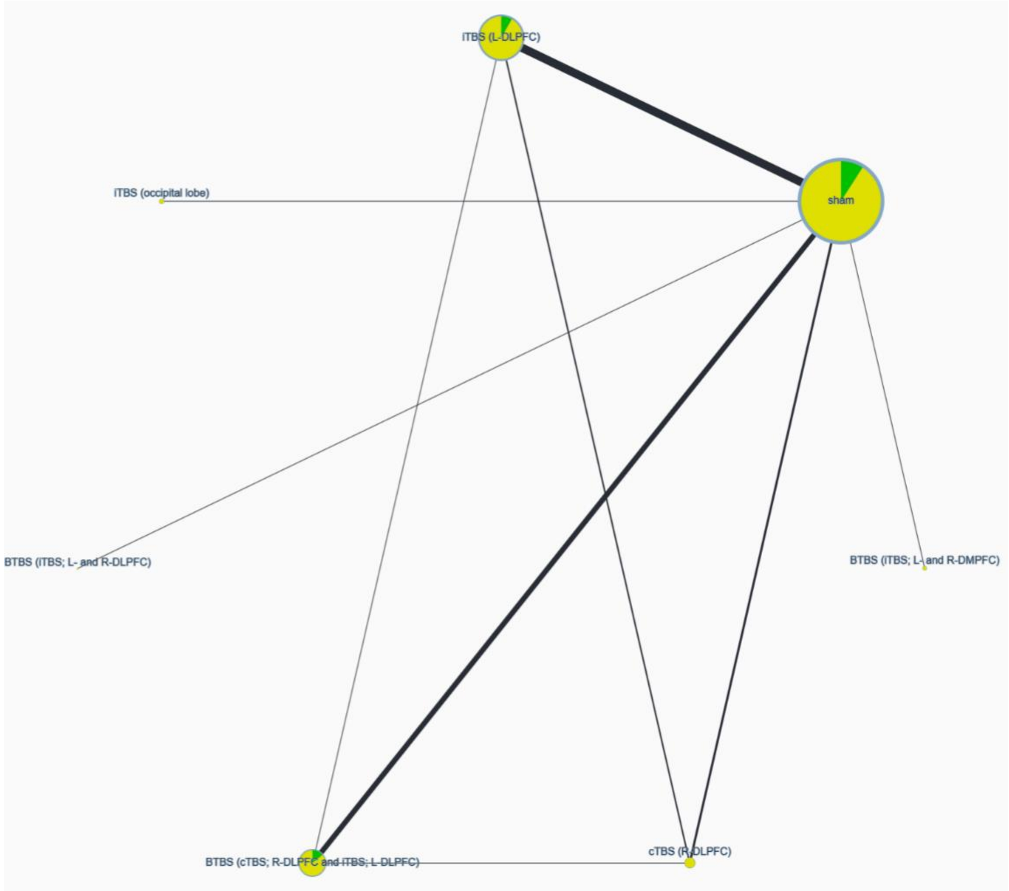

Node size by sample size

Node color by risk of bias

Green: low overall risk of bias

Yellow: moderate overall risk of bias

Edge width by number of studies

BTBS: bilateral theta burst stimulation

cTBS: continuous theta burst stimulation

iTBS: intermittent theta burst stimulation

BTBS: bilateral theta burst stimulation

L-DLPFC: left-dorsolateral prefrontal cortex

L- and R-DMPFC: left- and right- dorsomedial prefrontal cortex

R-DLPFC: right-dorsolateral prefrontal cortex

League table (RR with 95% confidence interval)

BTBS (cTBS; R-DLPFC and iTBS; L-DLPFC)	**4.497 (1.011, 20.009)**	1.831 (0.480, 6.989)	1.551 (0.657, 3.663)	0.947 (0.492, 1.824)	0.178 (0.018, 1.753)	**1.897 (1.110, 3.244)**
	BTBS (iTBS; L- and R-DLPFC)	0.407 (0.064, 2.606)	0.345 (0.071, 1.668)	**0.211 (0.049, 0.909)**	**0.040 (0.003, 0.546)**	0.422 (0.105, 1.699)
		BTBS (iTBS; L- and R-DMPFC)	0.847 (0.202, 3.546)	0.517 (0.140, 1.909)	0.097 (0.008, 1.232)	1.036 (0.304, 3.536)
			cTBS (R-DLPFC)	0.611 (0.276, 1.350)	0.115 (0.011, 1.195)	1.224 (0.585, 2.559)
				iTBS (L-DLPFC)	0.188 (0.019, 1.814)	**2.003 (1.283, 3.126)**
					iTBS (occipital lobe)	**10.666 (1.154, 98.603)**
						Sham

Global heterogeneity

As previously suggested (Huhn 2019), the common τ^2 was compared to the empirical distributions of heterogeneity found in the meta-analyses of pharmacological treatments for mental health outcomes, with a median of the τ^2 distribution of 0.049 and an inter-quartile range of 0.010 to 0.242 (Rhodes 2015), and the heterogeneity was considered low when the estimated τ^2 was below the 25% quartile, moderate when between 25% and 50% of the quartile, and high when above the 50% quartile.

Huhn M, et al. Lancet 2019;394(10202):939-51

Rhodes KM, et al. J Clin Epidemiol 2015;68(1):52-60

Between study variance (τ^2): 0.213 (heterogeneity assessment: moderate to high)

Random-effects design-by-treatment interaction model

$p < 0.1$ was considered as considerable global inconsistency.

χ^2 statistic: 4.205 (4 degrees of freedom), P value: 0.379

Local heterogeneity (I^2) and incoherence (SIDE test)

$I^2 \geq 50\%$ was considered as considerable heterogeneity.

As a general rule, there are “no concerns” if the p-value is >0.10, independent of the position of the 95% CIs with respect to the range of equivalence, because the evidence for incoherence is weak ($p > 0.10$).

Nikolakopoulou A, et al., PLOS Medicine 2020 17 1-19, Papakonstantinou T, et al., Campbell Systematic Reviews 2020 16 e1080

	NMA RR	Direct RR	I^2	Indirect RR	P value (SIDE test)

BTBS (cTBS; R-DLPFC and iTBS; L-DLPFC) vs cTBS (R-DLPFC)	1.551 (0.657, 3.663)	3.333 (0.746, 14.886)	na	1.064 (0.372, 3.040)	0.221
BTBS (cTBS; R-DLPFC and iTBS; L-DLPFC) vs iTBS (L-DLPFC)	0.947 (0.492, 1.824)	1.667 (0.471, 5.902)	na	0.770 (0.358, 1.656)	0.306
BTBS (cTBS; R-DLPFC and iTBS; L-DLPFC) vs Sham	1.897 (1.110, 3.244)	1.745 (0.926, 3.286)	60.6%	3.605 (0.812, 16.008)	0.366
cTBS (R-DLPFC) vs iTBS (L-DLPFC)	0.611 (0.276, 1.350)	0.874 (0.270, 2.833)	na	0.453 (0.155, 1.326)	0.418
cTBS (R-DLPFC) vs Sham	1.224 (0.585, 2.559)	1.165 (0.489, 2.774)	0.0%	1.391 (0.342, 5.651)	0.833
iTBS (L-DLPFC) vs Sham	2.003 (1.283, 3.126)	2.290 (1.437, 3.649)	48.4%	0.649 (0.169, 2.486)	**0.081**

Funnel plot (only sham-controlled trials)

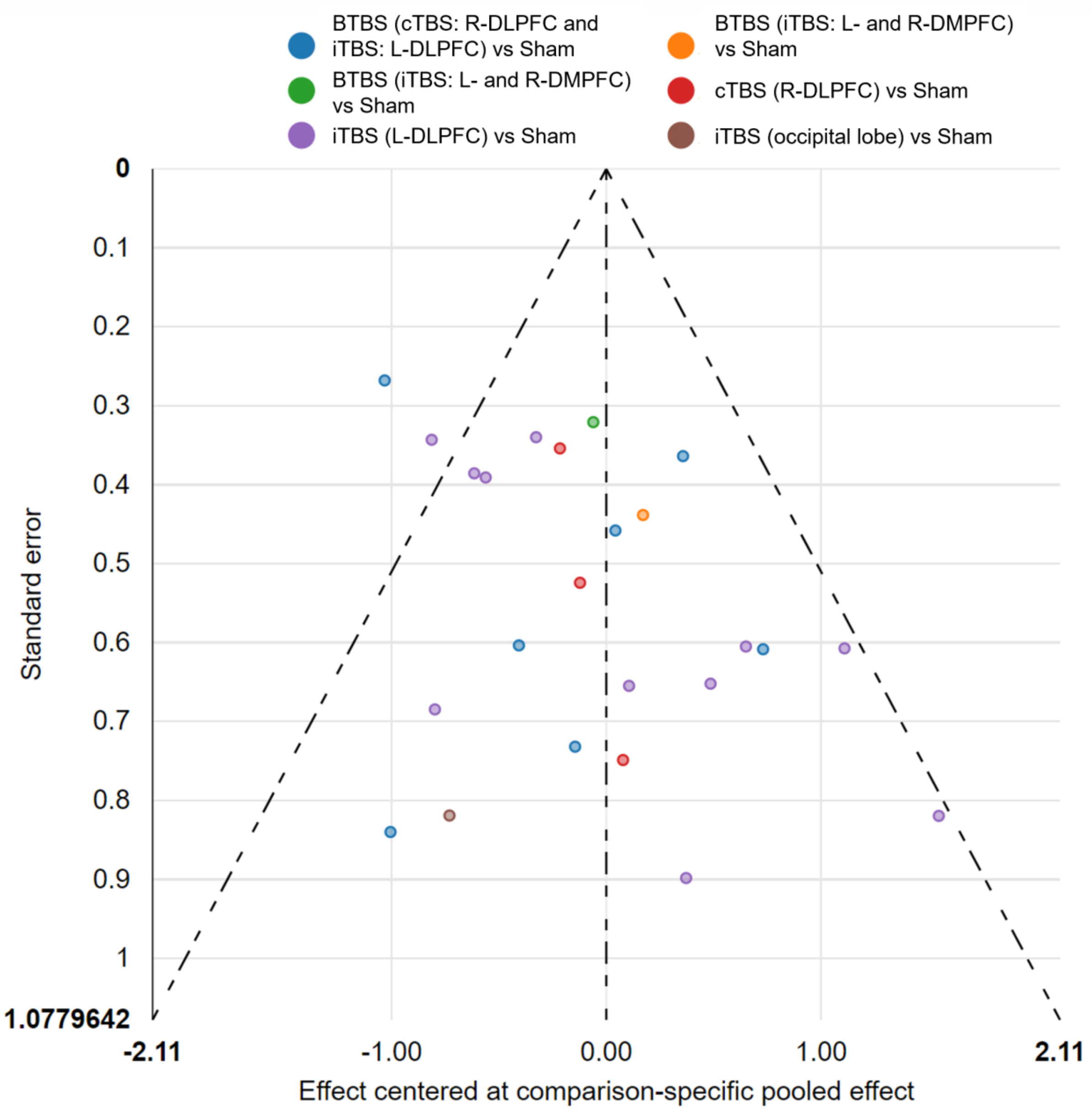

Meta-regression analysis (the sham was the control)

Potential moderators	β (SE)
Mean age (k = 23)	-0.285 (-1.258, 0.695)
Proportion of females (k = 23)	0.415 (-0.427, 1.274)
Total number of participants (k = 23)	-0.126 (-0.895, 0.706)
Minimum depressive symptoms at baseline (k = 23)	0.433 (-0.431, 1.360)
Diagnosis (k = 22)*	-0.771 (-1.595, 0.016)
Publication year (k = 23)	0.053 (-0.875, 1.000)
Overall risk of bias (k = 23) †	-0.170 (-1.626, 1.223)
Locating method (k = 23) ‡	0.035 (-0.837, 0.916)
Total number of sessions during the study (k = 23)	-0.423 (-1.203, 0.336)

* One study (Chistyakov 2010) did not report the MDD and BD proportion. If a study included both patients with MDD and BD, the diagnosis of more than 50% of the patients was adopted for the meta-regression (i.e., if a study had >50% individuals with MDD, the study is included in the MDD subgroup).

† Low overall risk of bias vs. some concerns risk of bias

‡ Using MRI vs. not using MRI

CINeMA confidence rating

CINeMA is a web application that simplifies the evaluation of confidence in the findings from a network meta-analysis. CINeMA is based on a methodological framework described in the following articles, which consider the following six domains: within-study bias, reporting bias, indirectness, imprecision, heterogeneity, and incoherence. CINeMA grades the confidence in the results of each treatment comparison as high, moderate, low, or very low. If the comparison had only indirect evidence, the comparison was downgraded one level.

Nikolakopoulou A, et al., PLOS Medicine 2020 17 1-19, Papakonstantinou T, et al., Campbell Systematic Reviews 2020 16 e1080

(1) Within-study bias: Risk of bias in RCTs for the main outcomes was assessed independently using the Cochrane risk-of-bias tool for randomized trials (RoB 2).

(2) Reporting bias: Comparison-adjusted funnel plots with less than 10 studies are not meaningful. Therefore, all comparisons other than iTBS (L-DLPFC) vs sham were "Suspected."

(3) Indirectness: No indirectness was assumed. Selected rule: Average

(4) Imprecision: For sham comparisons the clinically meaningful threshold was set at a risk ratio of higher or lower than 1. For sham comparisons the clinically meaningful threshold was set at a standardized mean difference of higher or lower than 0. For comparisons of two TBS treatments the clinically meaningful threshold was set at standardized mean differences of -0.1 and 0.1 for continuous outcomes and at a risk ratio of 0.8 and 1.25

for dichotomous outcomes.

(5) Heterogeneity: We used recommendations automatically provided by CINeMA.

(6) Incoherence: We used recommendations automatically provided by CINeMA.

Comparison	Number of studies	Within-study bias	Reporting bias	Indirectness	Imprecision	Heterogeneity	Incoherence	Confidence rating
BTBS (cTBS; R-DLPFC and iTBS; L-DLPFC) vs cTBS (R-DLPFC)	1	Some concerns	Some concerns	No concerns	Major concerns	No concerns	No concerns	Very low
BTBS (cTBS; R-DLPFC and iTBS; L-DLPFC) vs iTBS (L-DLPFC)	1	Some concerns	Some concerns	No concerns	Major concerns	No concerns	No concerns	Very low
BTBS (cTBS; R-DLPFC and iTBS; L-DLPFC) vs Sham	7	Some concerns	Some concerns	No concerns	No concerns	Major concerns	No concerns	Very low
BTBS (iTBS; L- and R-DLPFC) vs Sham	1	Some concerns	Some concerns	No concerns	Major concerns	No concerns	No concerns	Very low
BTBS (iTBS; L- and R-DMPFC) vs Sham	1	Some concerns	Some concerns	No concerns	Major concerns	No concerns	No concerns	Very low
cTBS (R-DLPFC) vs iTBS (L-DLPFC)	2	Some concerns	Some concerns	No concerns	Major concerns	No concerns	No concerns	Very low
cTBS (R-DLPFC) vs Sham	3	Some concerns	Some concerns	No concerns	Major concerns	No concerns	No concerns	Very low
iTBS (L-DLPFC) vs Sham	11	Some concerns	No concerns	No concerns	No concerns	Major concerns	Major concerns	Very low
iTBS (occipital lobe) vs Sham	1	Some concerns	Some concerns	No concerns	No concerns	Major concerns	No concerns	Very low
BTBS (cTBS; R-DLPFC and iTBS; L-DLPFC) vs BTBS (iTBS; L- and R-DLPFC)	0	Some concerns	Some concerns	No concerns	No concerns	Major concerns	No concerns	Very low
BTBS (cTBS; R-DLPFC and iTBS; L-DLPFC) vs BTBS (iTBS; L- and R-DMPFC)	0	Some concerns	Some concerns	No concerns	Major concerns	No concerns	No concerns	Very low
BTBS (cTBS; R-DLPFC and iTBS; L-DLPFC) vs iTBS (occipital lobe)	0	Some concerns	Some concerns	No concerns	Major concerns	No concerns	No concerns	Very low

BTBS (iTBS; L- and R-DLPFC) vs BTBS (iTBS; L- and R-DMPFC)	0	Some concerns	Some concerns	No concerns	Major concerns	No concerns	No concerns	Very low
BTBS (iTBS; L- and R-DLPFC) vs cTBS (R-DLPFC)	0	Some concerns	Some concerns	No concerns	Major concerns	No concerns	No concerns	Very low
BTBS (iTBS; L- and R-DLPFC) vs iTBS (L-DLPFC)	0	Some concerns	Some concerns	No concerns	No concerns	Major concerns	No concerns	Very low
BTBS (iTBS; L- and R-DLPFC) vs iTBS (occipital lobe)	0	Some concerns	Some concerns	No concerns	No concerns	No concerns	No concerns	Very low
BTBS (iTBS; L- and R-DMPFC) vs cTBS (R-DLPFC)	0	Some concerns	Some concerns	No concerns	Major concerns	No concerns	No concerns	Very low
BTBS (iTBS; L- and R-DMPFC) vs iTBS (L-DLPFC)	0	Some concerns	Some concerns	No concerns	Major concerns	No concerns	No concerns	Very low
BTBS (iTBS; L- and R-DMPFC) vs iTBS (occipital lobe)	0	Some concerns	Some concerns	No concerns	Major concerns	No concerns	No concerns	Very low
cTBS (R-DLPFC) vs iTBS (occipital lobe)	0	Some concerns	Some concerns	No concerns	Major concerns	No concerns	No concerns	Very low
iTBS (L-DLPFC) vs iTBS (occipital lobe)	0	Some concerns	Some concerns	No concerns	Major concerns	No concerns	No concerns	Very low

Pairwise meta-analysis for BTBS (cTBS; R-DLPFC and iTBS; L-DLPFC) vs. sham

7 studies, 288 participants

Forest plot

Study name	Statistics for each study					Risk ratio and 95% CI
	Risk ratio	Lower limit	Upper limit	Z-Value	p-Value	
Chou 2020	3.049	1.450	6.412	2.940	0.003	
Holczer 2021	1.909	0.383	9.508	0.789	0.430	
Li 2014	5.000	1.311	19.074	2.356	0.018	
Murga? 2023	0.750	0.101	5.544	-0.282	0.778	
Plewnia 2014	2.250	0.868	5.832	1.669	0.095	
Prasser 2015	1.417	0.395	5.080	0.535	0.593	
Tavares 2021	0.714	0.419	1.219	-1.235	0.217	
	1.745	0.926	3.286	1.723	0.085	

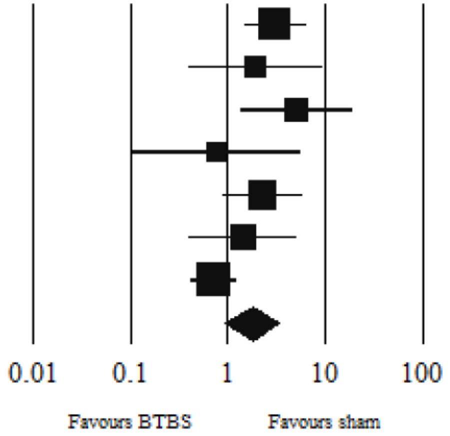

$I^2 = 66.59\%$

The univariable meta-regression analysis

	Active-sham difference				Active treatment			Sham treatment		
	Coefficient	95% CI	p	I^2 (%)	Coefficient	95% CI	p	Coefficient	95% CI	p
Diagnosis (studies including individuals with mixed state vs. studies not individuals with mixed state) (k = 7)	0.247	-1.773, 2.266	0.811	67.15	0.976	-0.965, 2.917	0.324	0.544	-1.022, 2.220	0.496
TRD (studies including individuals with TRD vs. studies including individuals with non TRD) (k = 7)	1.164	0.463, 1.864	**0.001**	0.00	1.374	0.655, 2.092	**0.001**	-0.417	-1.398, 0.563	0.404
Percent female (k =7)	-0.034	-0.116, 0.047	0.409	49.97	-0.023	-0.119, 0.073	0.638	0.036	-0.029, 0.101	0.280
Mean age (k = 7)	0.112	-0.019, 0.242	0.094	34.83	0.037	-0.142, 0.216	0.688	-0.131	-0.234, -0.029	**0.012**
Total number of participants (k = 7)	-0.009	-0.028, 0.011	0.390	44.78	0.005	-0.023, 0.033	0.726	0.015	0.003, 0.028	**0.016**
Studies that were used MRI vs. studies that were not used MRI (k =7)	-0.357	-1.773, 1.059	0.621	63.50	0.156	-1.368, 1.679	0.841	0.822	-0.293, 1.936	0.149
Studies that were used sham coil vs. studies that were not used sham coil (k = 7)	-0.468	-1.796, 0.859	0.489	60.03	-0.542	-1.963, 0.880	0.455	0.324	-0.839, 1.487	0.585
Percent motor threshold (k =7)	-0.008	-0.042, 0.026	0.641	66.26	0.002	-0.031, 0.035	0.905	0.014	-0.016, 0.044	0.359
Number of sessions during a trial (k =7)	-0.042	-0.118, 0.034	0.274	55.29	-0.029	-0.115, 0.057	0.508	0.045	-0.017, 0.108	0.155
Number of pulses during a session (k =7)	-0.000	-0.001, 0.001	0.414	43.17	-0.000	-0.000, 0.001	0.616	0.000	-0.000, 0.000	0.379
Number of pulses during a trial (k =7)	-0.000	-0.000, -0.000	**0.001**	0.00	-0.000	-0.000, 0.000	0.333	0.000	0.000, 0.000	**0.012**
Publication year (k =7)	-0.114	-0.287, 0.060	0.199	50.32	-0.094	-0.298, 0.111	0.369	0.117	-0.017, 0.251	0.087

95% CI: 95% confidence interval, N: number of studies

There was no study which compared BTBS (cTBS; R-DLPFC and iTBS; L-DLPFC) with sham for individuals with bipolar disorder. There was no study which compared accelerated BTBS (cTBS; R-DLPFC and iTBS; L-DLPFC) with sham. All studies included in the meta-regression analysis was evaluated as some concern for overall risk of bias.

Bubble plot: an association between the effect size for the response rate vs. studies including individuals with TRD or not

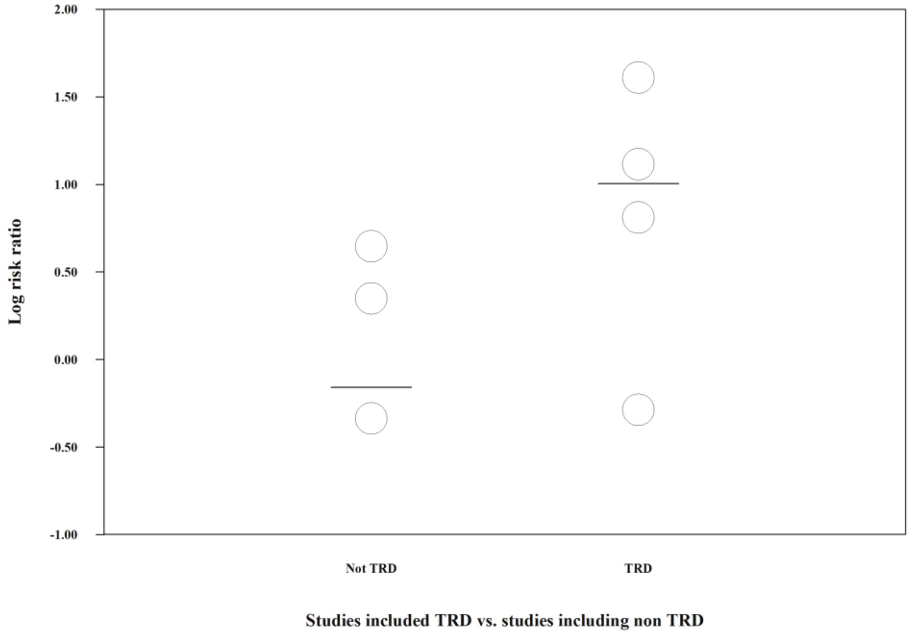

	Studies including individuals with TRD vs. studies including individuals with non TRD (K = 7)			
	Coefficient	95% CI	p	I^2 (%)
**Active-sham difference**	1.164	0.463, 1.864	**0.001**	0.00
**Active treatment**	1.374	0.655, 2.092	**0.001**	
**Sham treatment**	-0.417	-1.398, 0.563	0.404	

BTBS

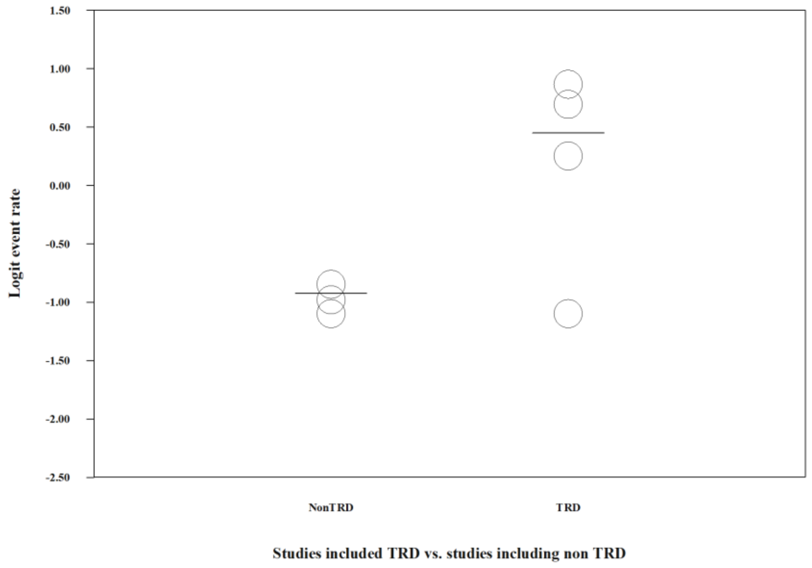

Sham

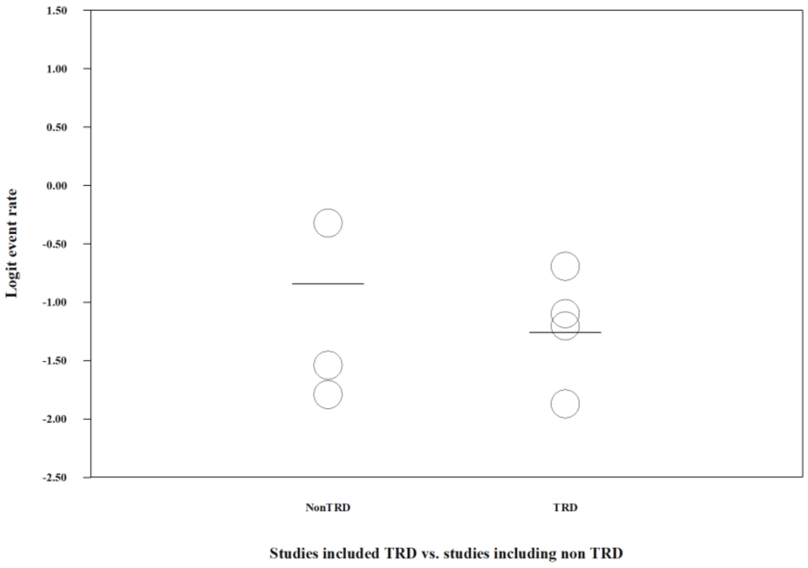

Bubble plot: the effect size for the response rate vs. the number of pulses during a trial

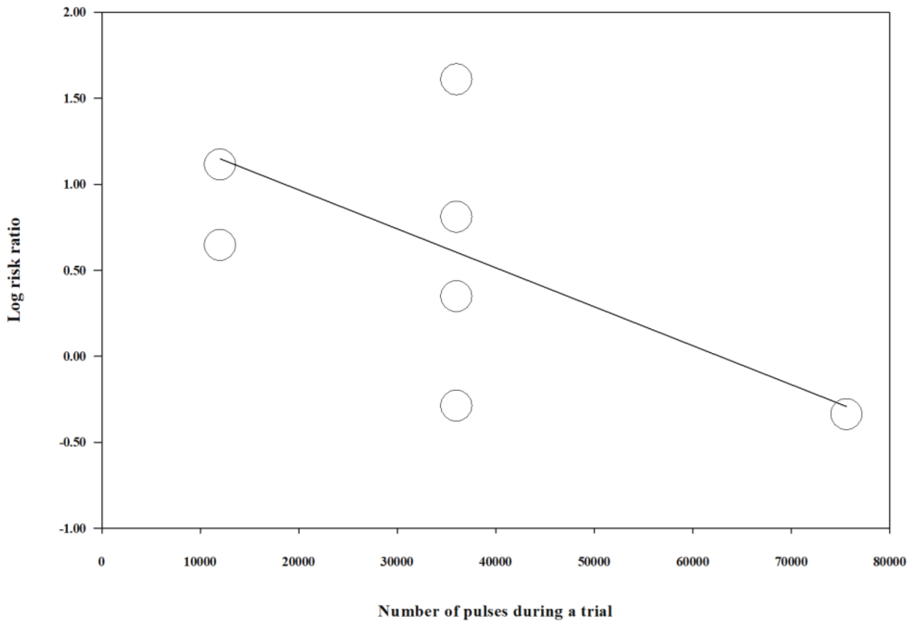

	Number of pulses during a trial (K = 7)			
	Coefficient	95% CI	p	I^2 (%)
**Active-sham difference**	-0.000	-0.000, -0.000	**0.001**	0.00
**Active treatment**	-0.000	-0.000, 0.000	0.333	
**Sham treatment**	0.000	0.000, 0.000	**0.012**	

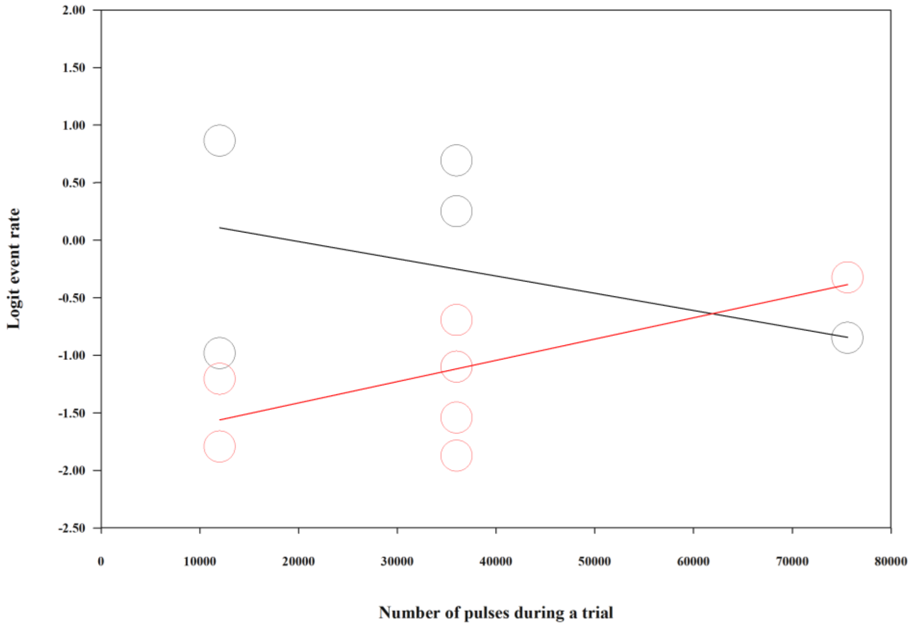

Black line, iTBS group; red line, sham group.

Pairwise meta-analysis for iTBS (L-DLPFC) vs. sham

11 studies, 476 participants

Forest plot

Study name	Risk ratio	Lower limit	Upper limit	Z-Value	p-Value
Beynel 2014	1.400	0.644	3.045	0.849	0.396
Bulteau 2019	1.750	0.880	3.480	1.596	0.111
Cole 2022	5.357	1.414	20.297	2.470	0.014
Duprat 2016	4.545	0.548	37.683	1.403	0.161
Li 2014	3.000	0.717	12.553	1.504	0.132
Li 2020	16.000	2.242	114.182	2.765	0.006
Li 2023	4.500	1.084	18.689	2.070	0.038
McGirr 2021	1.056	0.244	4.567	0.072	0.942
Wilkening 2022	1.278	0.587	2.782	0.618	0.537
Zavorotnyy 2020	1.038	0.520	2.074	0.107	0.915
Zhao 2023	8.609	2.257	32.839	3.151	0.002
	2.290	1.437	3.649	3.485	0.000

Risk ratio and 95% CI

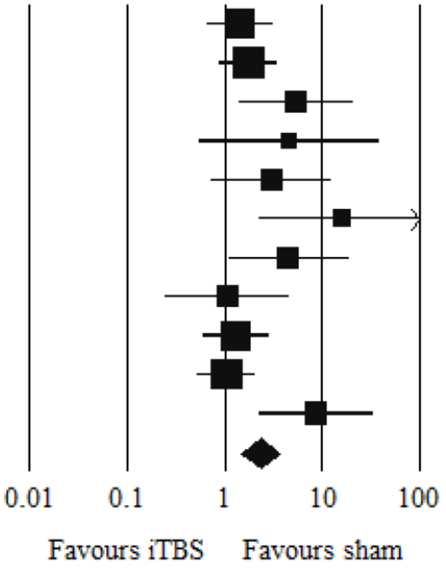

$I^2 = 48.41\%$

The univariable meta-regression analysis

	Active-sham difference				Active treatment			Sham treatment		
	Coefficient	95% CI	p	I^2 (%)	Coefficient	95% CI	p	Coefficient	95% CI	p
Diagnosis (studies including individuals with MDD vs. studies including individuals with BDep) (k = 11)	0.720	-0.305, 1.744	0.169	50.18	-0.357	-1.786, 1.071	0.624	-1.286	-2.562, -0.010	**0.048**
TRD (Studies including individuals with TRD vs. studies including individuals with non TRD) (k = 11)	0.388	-0.619, 1.390	0.450	49.17	-0.054	-1.351, 1.242	0.935	-0.345	-1.707, 1.017	0.620
Proportion of females (k =11)	0.030	-0.003, 0.063	0.071	39.02	-0.008	-0.051, 0.034	0.702	-0.044	-0.084, -0.003	**0.035**
Mean age (k = 11)	-0.027	-0.077, 0.022	0.280	49.31	-0.003	-0.066, 0.059	0.917	0.045	-0.016, 0.107	0.149
Total number of participants (k =11)	0.001	-0.020, 0.023	0.902	53.45	-0.024	-0.050, 0.002	0.066	-0.018	-0.047, 0.010	0.200
Studies that were used MRI vs. studies that were not used MRI (k =11)	0.431	-0.710, 1.573	0.459	53.48	0.535	-0.686, 1.756	0.390	-0.324	-1.800, 1.151	0.667
Studies that were used sham coil vs. studies that were not used sham coil (k = 11)	0.373	-0.861, 1.601	0.553	52.59	0.733	-0.652, 2.118	0.300	0.061	-1.563, 1.684	0.942
Percent motor threshold (k =11)	-0.022	-0.057, 0.012	0.205	47.78	-0.048	-0.078, -0.019	**0.001**	-0.010	-0.054, 0.034	0.665
Number of sessions during a day (k =11)	0.053	-0.150, 0.256	0.607	52.00	0.032	-0.198, 0.262	0.786	-0.043	-0.298, 0.212	0.743
Number of sessions during a trial (k =11)	-0.025	-0.064, 0.014	0.210	45.21	0.018	-0.029, 0.066	0.448	0.046	0.002, 0.090	**0.042**
Number of pulses during a session (k =11)	0.001	0.000, 0.002	**0.007**	20.07	0.000	-0.001, 0.001	0.720	-0.001	-0.002, -0.000	**0.010**
Studies with 600 pulses in a session vs. studies with >600 pulses in a session studies (k =11)	0.965	-0.094, 2.025	0.074	39.09	0.945	-0.529, 2.419	0.209	-0.651	-2.151, 0.849	0.395
Studies with $\geq$1000 pulses in a session vs. studies with <1000 pulses in a session studies (k =11)	0.988	0.220, 1.756	**0.012**	25.91	-0.143	-1.420, 1.133	0.825	-1.472	-2.314, -0.631	**0.001**
Number of pulses during a day (k =11)	0.000	-0.000, 0.000	0.354	48.86	0.000	-0.000, 0.000	0.780	-0.000	-0.000, 0.000	0.446
Number of pulses during a trial (k =11)	0.000	-0.000, 0.000	0.499	50.63	0.000	-0.000, 0.000	0.354	-0.000	-0.000, 0.000	0.965
aiTBS studies vs. once daily iTBS studies (k =11)	0.828	-0.159, 1.814	0.100	38.44	0.031	-1.175, 1.238	0.960	-1.043	-2.242, 0.157	0.089
Intersession interval (k = 7)*	-0.001	-0.007, 0.005	0.784	38.40	0.012	0.003, 0.020	**0.006**	0.010	0.000, 0.018	**0.046**
Publication year (k =11)	0.059	-0.095, 0.212	0.455	51.60	0.027	-0.166, 0.220	0.784	-0.099	-0.298, 0.100	0.329
Overall risk of bias (low vs some concerns) (k = 11)	-0.084	-1.440, 1.271	0.903	52.57	0.143	-1.415, 1.702	0.857	0.279	-1.352, 1.911	0.737

95% CI: 95% confidence interval, N: number of studies

*The meta-regression analysis included only accelerated iTBS studies

Bubble plot: an association between the effect size for the response rate and number of pulses during a session

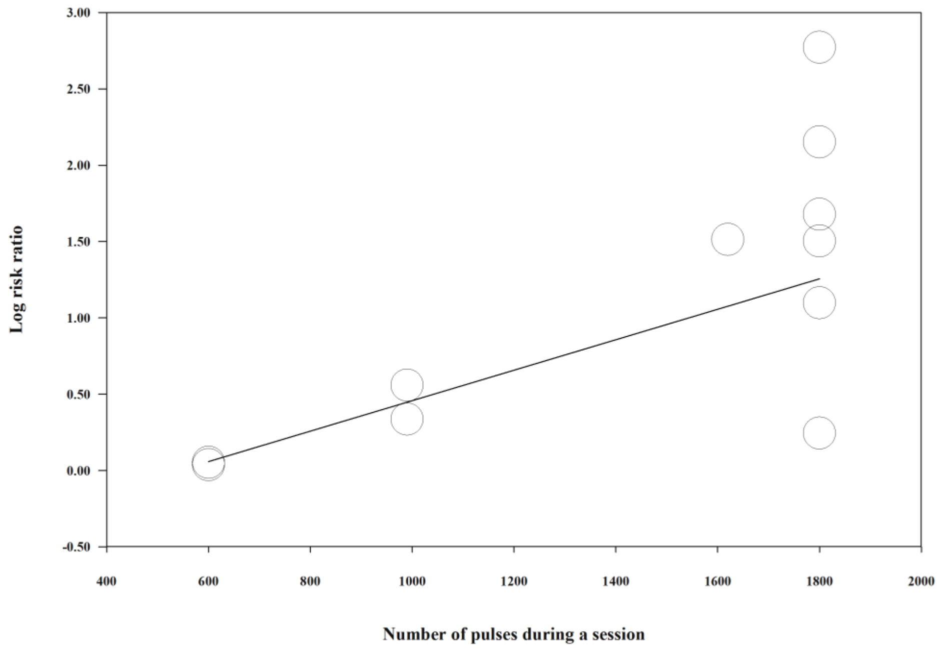

	Number of pulses during a session (K =11)			
	Coefficient	95% CI	p	I^2 (%)
**Active-sham difference**	0.001	0.000, 0.002	**0.007**	20.07
**Active treatment**	0.000	-0.001, 0.001	0.720	
**Sham treatment**	-0.001	-0.002, -0.000	**0.010**	

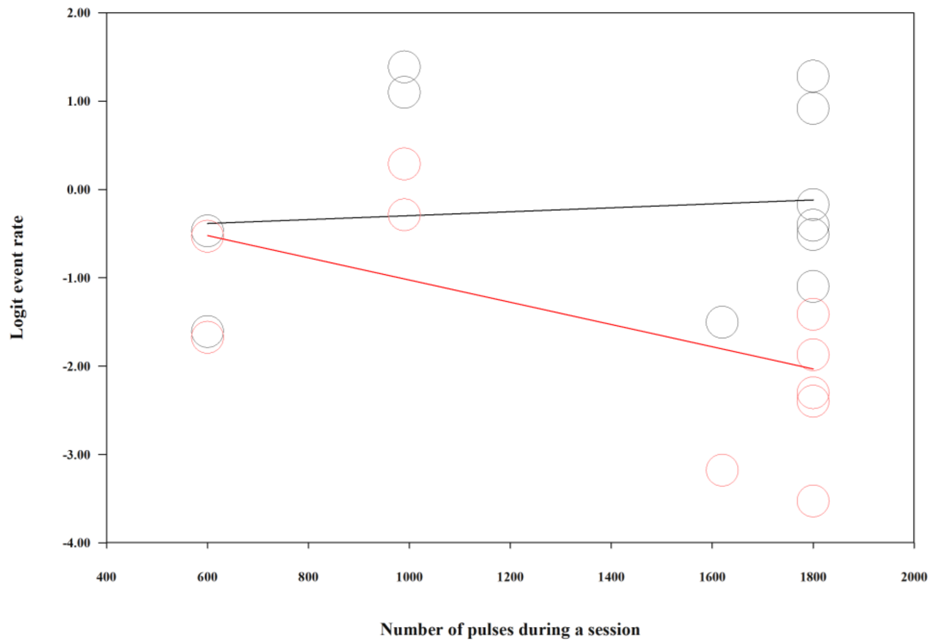

Black line, iTBS group; red line, sham group.

Bubble plot: an association between the effect size for the response rate and studies with ≥1000 pulses in a session vs. studies with <1000 pulses in a session

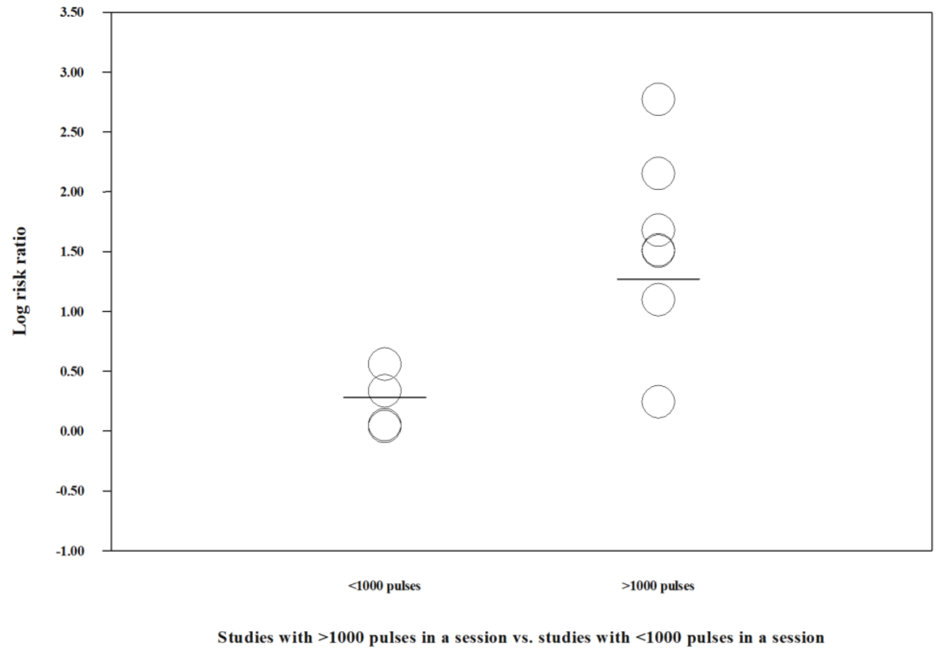

	**Studies with >1000 pulses in a session vs. studies with <1000 pulses in a session studies (K =11)**			
	Coefficient	95% CI	p	I^2 (%)
**Active-sham difference**	0.988	0.220, 1.756	0.012	25.91
**Active treatment**	-0.143	-1.420, 1.133	0.825	
**Sham treatment**	-1.472	-2.314, -0.631	0.001	

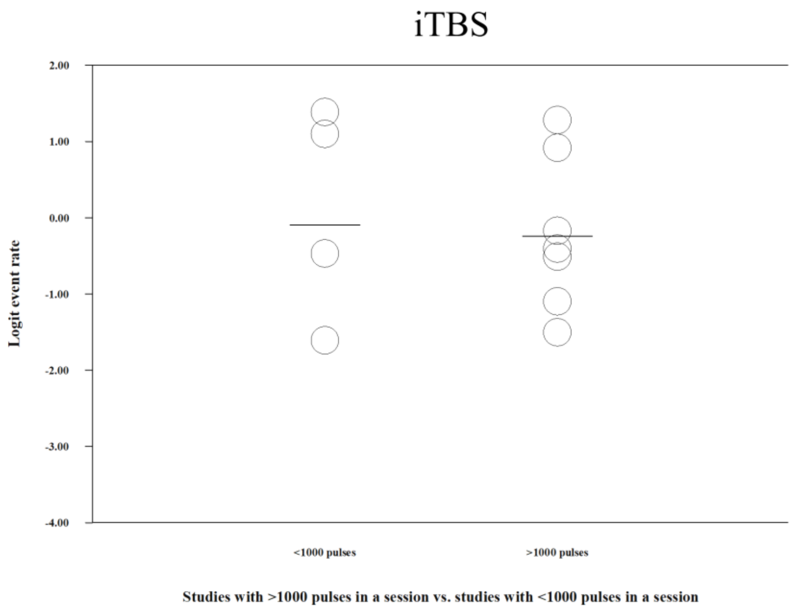

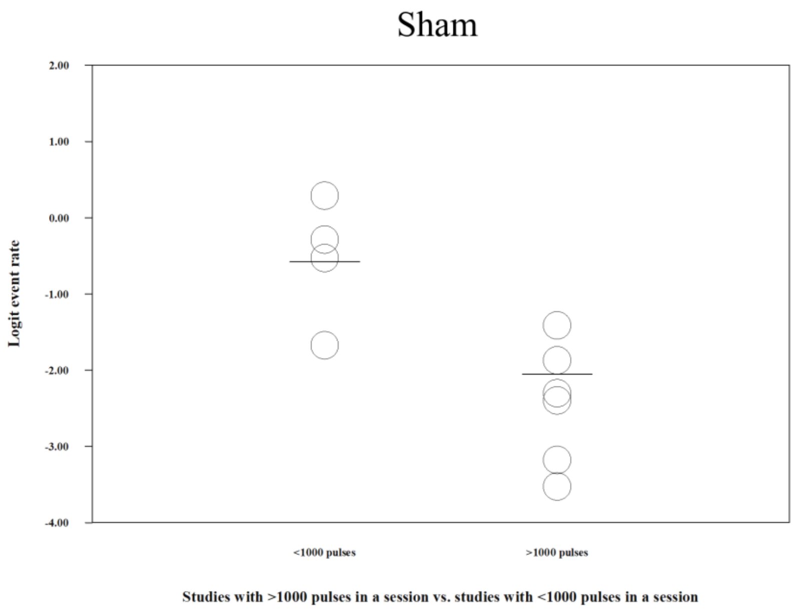

Appendix S2. Depression symptom improvement

Network meta-analysis

23 studies, 924 participants

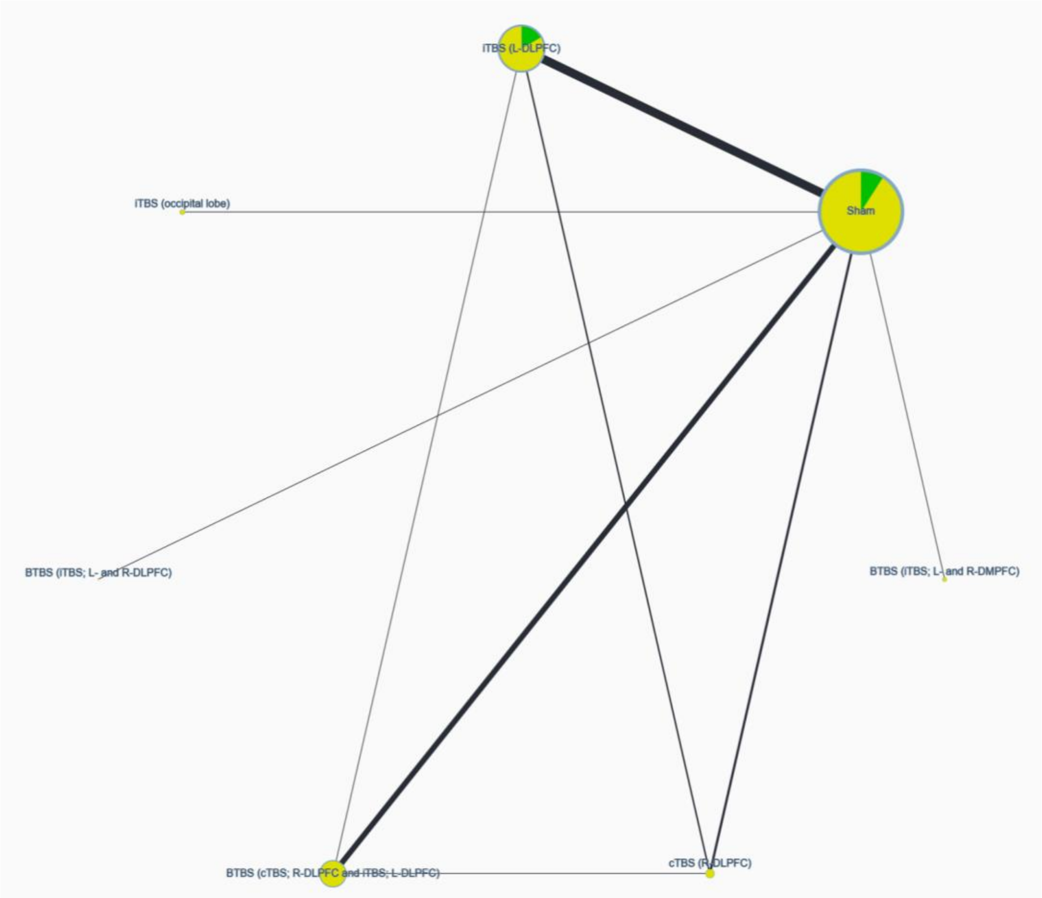

League table (SMD with 95% confidence interval)

BTBS (cTBS; R-DLPFC and iTBS; L-DLPFC)	**-2.079 (-3.828, -0.330)**	-1.238 (-2.815, 0.338)	**-0.903 (-1.791, -0.014)**	-0.322 (-0.997, 0.352)	-0.541 (-2.048, 0.966)	**-0.947 (-1.502, -0.392)**
	BTBS (iTBS; L- and R-DLPFC)	0.841 (-1.379, 3.060)	1.176 (-0.641, 2.994)	**1.757 (0.044, 3.470)**	1.538 (-0.633, 3.709)	1.132 (-0.527, 2.790)
		BTBS (iTBS; L- and R-DMPFC)	0.336 (-1.317, 1.988)	0.916 (-0.620, 2.452)	0.697 (-1.338, 2.732)	0.291 (-1.184, 1.766)
			cTBS (R-DLPFC)	0.580 (-0.209, 1.370)	0.362 (-1.225, 1.948)	-0.045 (-0.789, 0.699)
				iTBS (L-DLPFC)	-0.219 (-1.684, 1.246)	**-0.625 (-1.052, -0.198)**
					iTBS (occipital lobe)	-0.406 (-1.808, 0.995)
						Sham

Global heterogeneity

Between study variance (τ^2): 0.431 (heterogeneity assessment: high)

Random-effects design-by-treatment interaction model

χ 2 statistic: 4.294 (4 degrees of freedom), P value: 0.368

Local heterogeneity (I^2) and incoherence (SIDE test)

	NMA SMD	Direct SMD	I^2	Indirect SMD	P value (SIDE test)
BTBS (cTBS; R-DLPFC and iTBS; L-DLPFC) vs cTBS (R-DLPFC)	-0.903 (-1.791, -0.014)	-1.946 (-3.510, -0.382)	na	-0.405 (-1.485, 0.675)	0.112
BTBS (cTBS; R-DLPFC and iTBS; L-DLPFC) vs iTBS (L-DLPFC)	-0.322 (-0.997, 0.352)	-0.662 (-2.145, 0.822)	na	-0.234 (-0.991, 0.523)	0.615
BTBS (cTBS; R-DLPFC and iTBS; L-DLPFC) vs Sham	-0.947 (-1.502, -0.392)	-0.953 (-1.525, -0.382)	81.6%	-0.850 (-3.163, 1.463)	0.933
cTBS (R-DLPFC) vs iTBS (L-DLPFC)	0.580 (-0.209, 1.370)	0.660 (-0.466, 1.786)	na	0.503 (-0.604, 1.610)	0.846
cTBS (R-DLPFC) vs Sham	-0.045 (-0.789, 0.699)	-0.159 (-1.030, 0.711)	0.0%	0.266 (-1.166, 1.698)	0.619
iTBS (L-DLPFC) vs Sham	-0.625 (-1.052, -0.198)	-0.706 (-1.150, -0.261)	83.2%	0.384 (-1.189, 1.957)	0.191

CINeMA confidence rating

Comparison	Number of studies	Within-study bias	Reporting bias	Indirectness	Imprecision	Heterogeneity	Incoherence	Confidence rating
BTBS (cTBS; R-DLPFC and iTBS; L-DLPFC) vs Sham	7	Some concerns	Some concerns	No concerns	No concerns	Major concerns	No concerns	Very low
BTBS (cTBS; R-DLPFC and iTBS; L-DLPFC) vs cTBS (R-DLPFC)	1	Some concerns	Some concerns	No concerns	No concerns	Major concerns	No concerns	Very low
BTBS (cTBS; R-DLPFC and iTBS; L-DLPFC) vs iTBS (L-DLPFC)	1	Some concerns	Some concerns	No concerns	Major concerns	No concerns	No concerns	Very low
BTBS (iTBS; L- and R-DLPFC) vs Sham	1	Some concerns	Some concerns	No concerns	Major concerns	No concerns	No concerns	Very low
BTBS (iTBS; L- and R-DMPFC) vs Sham	1	Some concerns	Some concerns	No concerns	Major concerns	No concerns	No concerns	Very low
cTBS (R-DLPFC) vs Sham	3	Some concerns	Some concerns	No concerns	Major concerns	No concerns	No concerns	Very low
iTBS (L-DLPFC) vs Sham	11	Some concerns	No concerns	No concerns	No concerns	Major concerns	No concerns	Low
iTBS (occipital lobe) vs Sham	1	Some concerns	Some concerns	No concerns	Major concerns	No concerns	No concerns	Very low
cTBS (R-DLPFC) vs iTBS (L-DLPFC)	2	Some concerns	Some concerns	No concerns	Major concerns	No concerns	No concerns	Very low
BTBS (cTBS; R-DLPFC and iTBS; L-DLPFC) vs BTBS (iTBS; L- and R-DLPFC)	0	Some concerns	Some concerns	No concerns	No concerns	Major concerns	No concerns	Very low
BTBS (cTBS; R-DLPFC and iTBS; L-DLPFC) vs BTBS (iTBS; L- and R-DMPFC)	0	Some concerns	Some concerns	No concerns	Major concerns	No concerns	No concerns	Very low
BTBS (cTBS; R-DLPFC and iTBS; L-DLPFC) vs iTBS (occipital lobe)	0	Some concerns	Some concerns	No concerns	Major concerns	No concerns	No concerns	Very low
BTBS (iTBS; L- and R-DLPFC) vs BTBS (iTBS; L- and R-DMPFC)	0	Some concerns	Some concerns	No concerns	Major concerns	No concerns	No concerns	Very low
BTBS (iTBS; L- and R-DLPFC) vs cTBS (R-DLPFC)	0	Some concerns	Some concerns	No concerns	Major concerns	No concerns	No concerns	Very low
BTBS (iTBS; L- and R-DLPFC) vs iTBS (L-DLPFC)	0	Some concerns	Some concerns	No concerns	No concerns	Major concerns	No concerns	Very low
BTBS (iTBS; L- and R-DLPFC) vs iTBS (occipital lobe)	0	Some concerns	Some concerns	No concerns	Major concerns	No concerns	No concerns	Very low
BTBS (iTBS; L- and R-DMPFC) vs cTBS (R-DLPFC)	0	Some concerns	Some concerns	No concerns	Major concerns	No concerns	No concerns	Very low
BTBS (iTBS; L- and R-DMPFC) vs iTBS (L-DLPFC)	0	Some concerns	Some concerns	No concerns	Major concerns	No concerns	No concerns	Very low
BTBS (iTBS; L- and R-DMPFC) vs iTBS (occipital lobe)	0	Some concerns	Some concerns	No concerns	Major concerns	No concerns	No concerns	Very low
cTBS (R-DLPFC) vs iTBS (occipital lobe)	0	Some concerns	Some concerns	No concerns	Major concerns	No concerns	No concerns	Very low
iTBS (L-DLPFC) vs iTBS (occipital lobe)	0	Some concerns	Some concerns	No concerns	Major concerns	No concerns	No concerns	Very low

Appendix S3. Remission rate

Network meta-analysis

15 studies, 716 participants

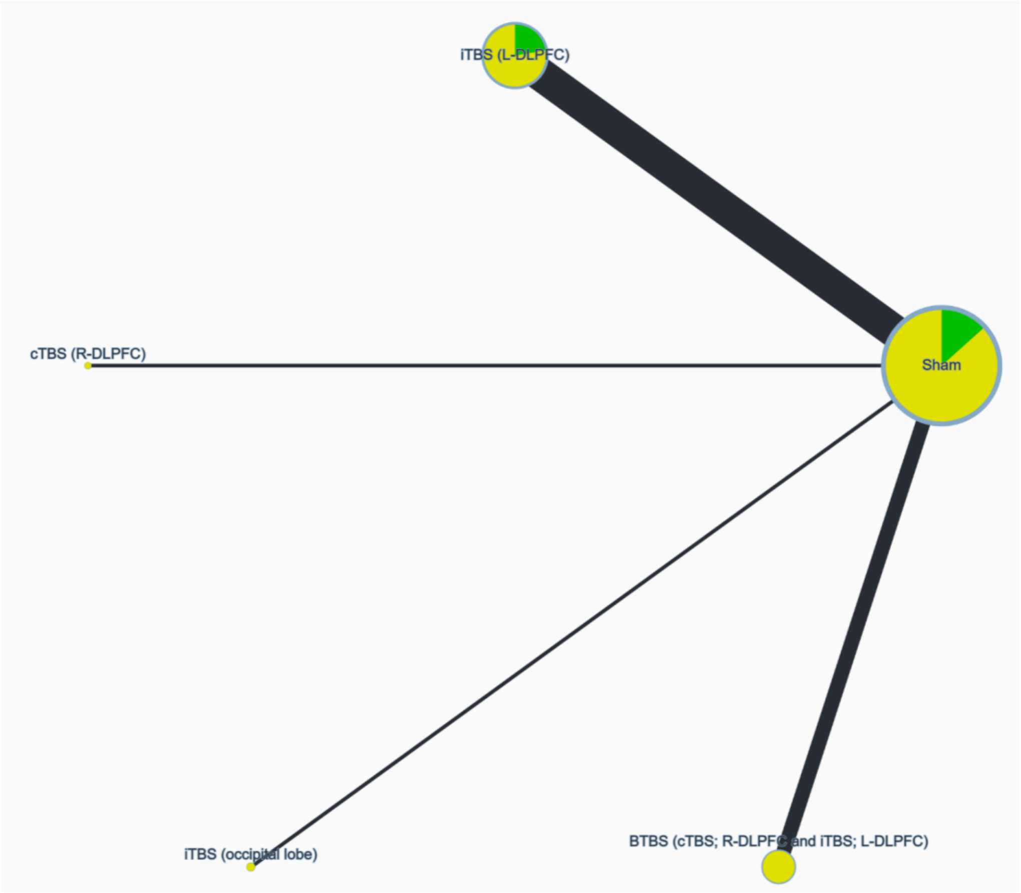

League table (RR with 95% confidence interval)

BTBS (cTBS; R-DLPFC and iTBS; L-DLPFC)	1.012 (0.089, 11.532)	0.734 (0.217, 2.485)	0.594 (0.016, 21.876)	1.587 (0.611, 4.121)
	cTBS (R-DLPFC)	0.725 (0.068, 7.709)	0.587 (0.009, 36.705)	1.568 (0.167, 14.704)
		iTBS (L-DLPFC)	0.809 (0.023, 28.443)	**2.162 (1.012, 4.618)**
			iTBS (occipital lobe)	2.673 (0.082, 86.591)
				Sham

Global heterogeneity

Between study variance (τ^2): 0.560 (heterogeneity assessment: high)

Random-effects design-by-treatment interaction model

na

Local heterogeneity (I^2) and incoherence (SIDE test)

	NMA RR	Direct RR	I^2	Indirect RR	P value (SIDE test)
BTBS (cTBS; R-DLPFC and iTBS; L-DLPFC) vs Sham	na	1.587 (0.611, 4.121)	58.3%	na	na
cTBS (R-DLPFC) vs Sham	na	1.568 (0.167, 14.704)	na	na	na
iTBS (L-DLPFC) vs Sham	na	2.162 (1.012, 4.618)	50.2%	na	na
iTBS (occipital lobe) vs Sham	na	2.673 (0.082, 86.591)	na	na	na

CINeMA confidence rating

Comparison	Number of studies	Within-study bias	Reporting bias	Indirectness	Imprecision	Heterogeneity	Incoherence	Confidence rating
BTBS (cTBS; R-DLPFC and iTBS; L-DLPFC) vs Sham	4	Some concerns	Some concerns	No concerns	Major concerns	No concerns	Major concerns	Very low
cTBS (R-DLPFC) vs Sham	1	Some concerns	Some concerns	No concerns	Major concerns	No concerns	Major concerns	Very low
iTBS (L-DLPFC) vs Sham	9	Some concerns	Some concerns	No concerns	No concerns	Major concerns	Major concerns	Very low
iTBS (occipital lobe) vs Sham	1	Some concerns	Some concerns	No concerns	Major concerns	No concerns	Major concerns	Very low
BTBS (cTBS; R-DLPFC and iTBS; L-DLPFC) vs cTBS (R-DLPFC)	0	Some concerns	Some concerns	No concerns	Major concerns	No concerns	Major concerns	Very low
BTBS (cTBS; R-DLPFC and iTBS; L-DLPFC) vs iTBS (L-DLPFC)	0	Some concerns	Some concerns	No concerns	Major concerns	No concerns	Major concerns	Very low
BTBS (cTBS; R-DLPFC and iTBS; L-DLPFC) vs iTBS (occipital lobe)	0	Some concerns	Some concerns	No concerns	Major concerns	No concerns	Major concerns	Very low
cTBS (R-DLPFC) vs iTBS (L-DLPFC)	0	Some concerns	Some concerns	No concerns	Major concerns	No concerns	Major concerns	Very low
cTBS (R-DLPFC) vs iTBS (occipital lobe)	0	Some concerns	Some concerns	No concerns	Major concerns	No concerns	Major concerns	Very low
iTBS (L-DLPFC) vs iTBS (occipital lobe)	0	Some concerns	Some concerns	No concerns	Major concerns	No concerns	Major concerns	Very low

Appendix S4. All-cause discontinuation

<u>Network meta-analysis</u>

17 studies, 768 participants

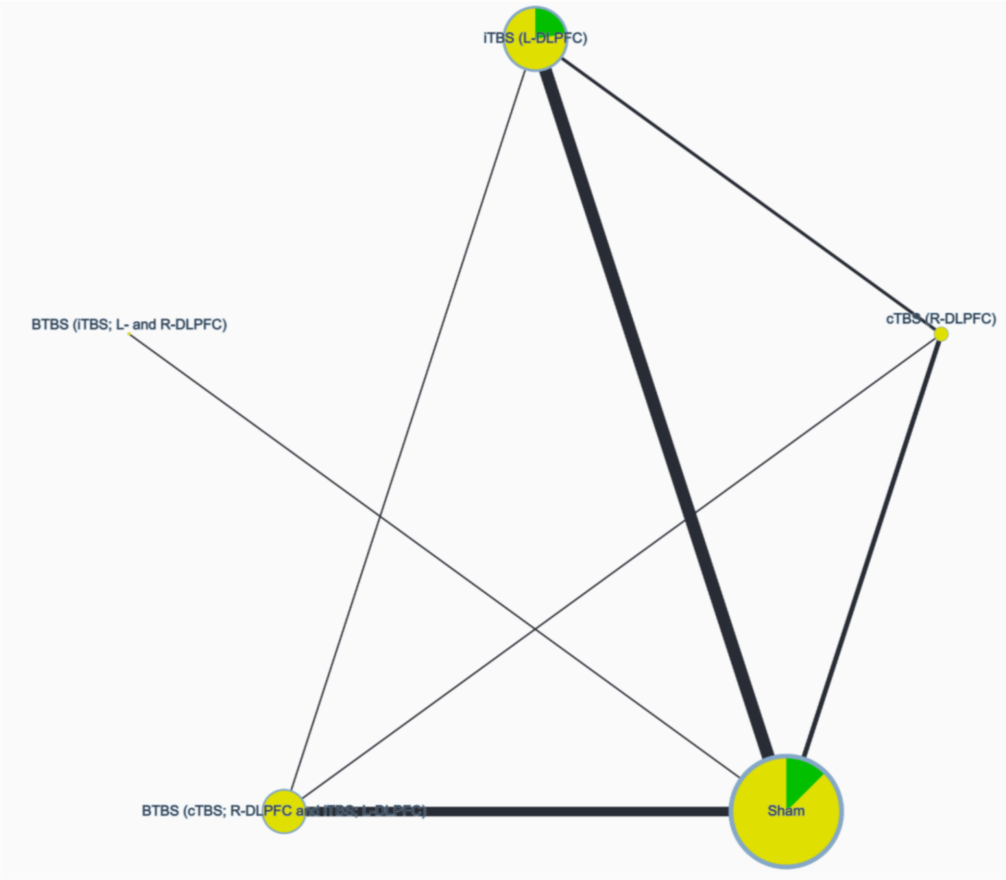

League table (risk ratio with 95% confidence interval)

BTBS (cTBS; R-DLPFC and iTBS; L-DLPFC)	0.274 (0.015, 5.165)	1.738 (0.350, 8.629)	1.156 (0.465, 2.875)	1.239 (0.817, 1.881)
	BTBS (iTBS; L- and R-DLPFC)	6.341 (0.234, 171.520)	4.220 (0.206, 86.392)	4.524 (0.247, 82.773)
		cTBS (R-DLPFC)	0.665 (0.126, 3.505)	0.713 (0.150, 3.385)
			iTBS (L-DLPFC)	1.072 (0.474, 2.424)
				Sham

Global heterogeneity

Between study variance (τ^2): 0.000 (heterogeneity assessment: low)

Random-effects design-by-treatment interaction model

χ 2 statistic: 0.279 (4 degrees of freedom), P value: 0.991

Local heterogeneity (I^2) and incoherence (SIDE test)

	NMA RR	Direct RR	I^2	Indirect RR	P value (SIDE test)
BTBS (cTBS; R-DLPFC and iTBS; L-DLPFC) vs cTBS (R-DLPFC)	1.738 (0.350, 8.629)	1.000 (0.021, 47.285)	na	1.950 (0.335, 11.358)	0.757
BTBS (cTBS; R-DLPFC and iTBS; L-DLPFC) vs iTBS (L-DLPFC)	1.156 (0.465, 2.875)	1.000 (0.021, 47.285)	na	1.166 (0.457, 2.978)	0.940
BTBS (cTBS; R-DLPFC and iTBS; L-DLPFC) vs Sham	1.239 (0.817, 1.881)	1.243 (0.818, 1.889)	0.0%	0.718 (0.002, 236.465)	0.853
cTBS (R-DLPFC) vs iTBS (L-DLPFC)	0.665 (0.126, 3.505)	1.076 (0.072, 15.978)	na	0.496 (0.060, 4.088)	0.658
cTBS (R-DLPFC) vs Sham	0.713 (0.150, 3.385)	0.591 (0.100, 3.501)	0.0%	1.322 (0.053, 33.069)	0.668
iTBS (L-DLPFC) vs Sham	1.072 (0.474, 2.424)	1.106 (0.479, 2.557)	0.0%	0.601 (0.017, 21.663)	0.745

CINeMA confidence rating

Comparison	Number of studies	Within-study bias	Reporting bias	Indirectness	Imprecision	Heterogeneity	Incoherence	Confidence rating
BTBS (cTBS; R-DLPFC and iTBS; L-DLPFC) vs Sham	6	Some concerns	Some concerns	No concerns	Major concerns	No concerns	No concerns	Very low
BTBS (cTBS; R-DLPFC and iTBS; L-DLPFC) vs cTBS (R-DLPFC)	1	Some concerns	Some concerns	No concerns	Major concerns	No concerns	No concerns	Very low
BTBS (cTBS; R-DLPFC and iTBS; L-DLPFC) vs iTBS (L-DLPFC)	1	Some concerns	Some concerns	No concerns	Major concerns	No concerns	No concerns	Very low
BTBS (iTBS; L- and R-DLPFC) vs Sham	1	Some concerns	Some concerns	No concerns	Major concerns	No concerns	No concerns	Very low
cTBS (R-DLPFC) vs Sham	3	Some concerns	Some concerns	No concerns	Major concerns	No concerns	No concerns	Very low
iTBS (L-DLPFC) vs Sham	8	Some concerns	No concerns	No concerns	Major concerns	No concerns	No concerns	Low
cTBS (R-DLPFC) vs iTBS (L-DLPFC)	2	Some concerns	Some concerns	No concerns	Major concerns	No concerns	No concerns	Very low
BTBS (cTBS; R-DLPFC and iTBS; L-DLPFC) vs BTBS (iTBS; L- and R-DLPFC)	0	Some concerns	Some concerns	No concerns	Major concerns	No concerns	No concerns	Very low
BTBS (iTBS; L- and R-DLPFC) vs cTBS (R-DLPFC)	0	Some concerns	Some concerns	No concerns	Major concerns	No concerns	No concerns	Very low
BTBS (iTBS; L- and R-DLPFC) vs iTBS (L-DLPFC)	0	Some concerns	Some concerns	No concerns	Major concerns	No concerns	No concerns	Very low

Appendix S5. Switch to mania

Network meta-analysis

7 studies, 395 participants

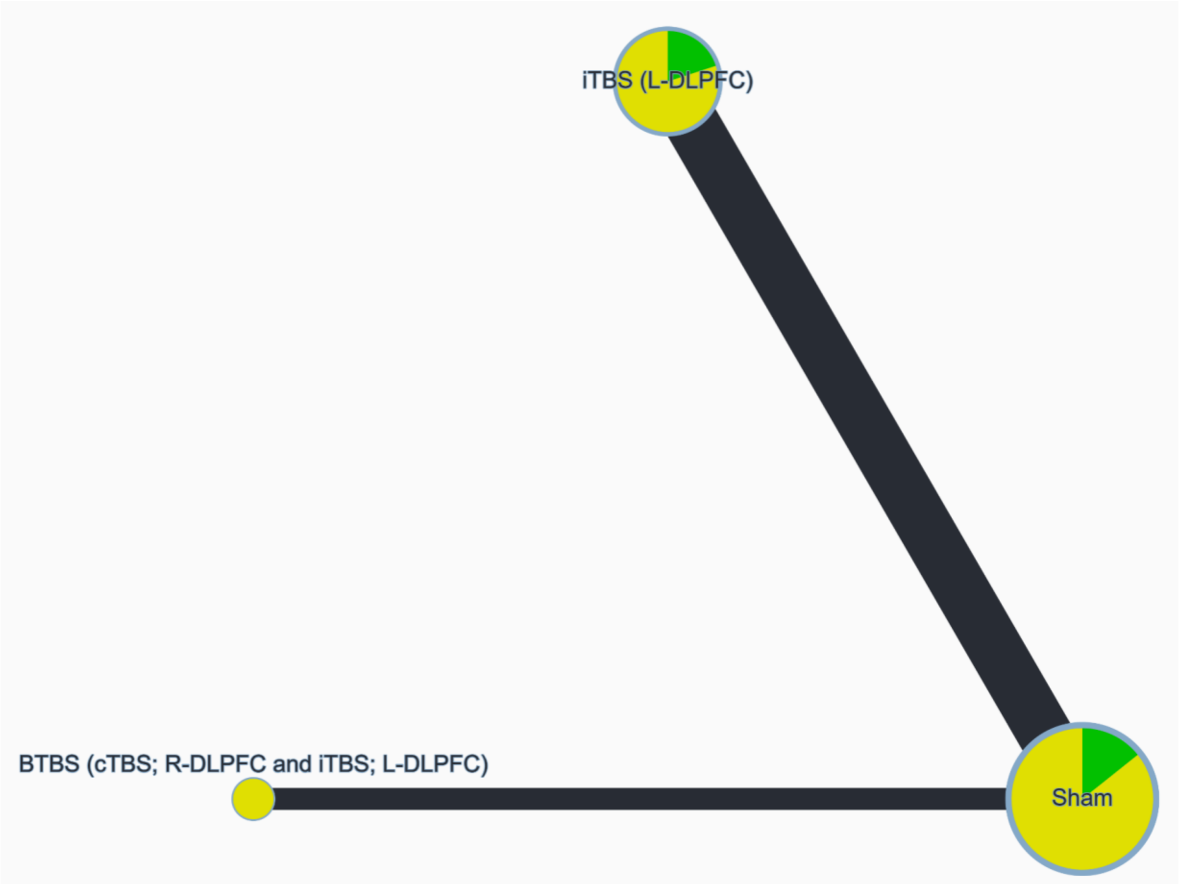

League table (RR with 95% confidence interval)

BTBS (cTBS; R-DLPFC and iTBS; L-DLPFC)	3.079 (0.203, 46.707)	4.368 (0.503, 37.944)
	iTBS (L-DLPFC)	1.419 (0.273, 7.384)
		Sham

Global heterogeneity

Between study variance (τ^2): 0.000 (heterogeneity assessment: low)

Random-effects design-by-treatment interaction model

na

Local heterogeneity (I^2) and incoherence (SIDE test)

	NMA RR	Direct RR	I^2	Indirect RR	P value (SIDE test)
BTBS (cTBS; R-DLPFC and iTBS; L-DLPFC) vs Sham	na	4.368 (0.503, 37.944)	0.0%	na	na
iTBS (L-DLPFC) vs Sham	na	1.419 (0.273, 7.384)	0.0%	na	na

CINeMA confidence rating

Comparison	Number of studies	Within-study bias	Reporting bias	Indirectness	Imprecision	Heterogeneity	Incoherence	Confidence rating
BTBS (cTBS; R-DLPFC and iTBS; L-DLPFC) vs Sham	2	Some concerns	Some concerns	No concerns	Major concerns	No concerns	Major concerns	Very low
iTBS (L-DLPFC) vs Sham	5	Some concerns	Some concerns	No concerns	Major concerns	No concerns	Major concerns	Very low
BTBS (cTBS; R-DLPFC and iTBS; L-DLPFC) vs iTBS (L-DLPFC)	0	Some concerns	Some concerns	No concerns	Major concerns	No concerns	Major concerns	Very low

Appendix S6. Headache/discomfort at treatment site

Network meta-analysis

10 studies, 340 participants

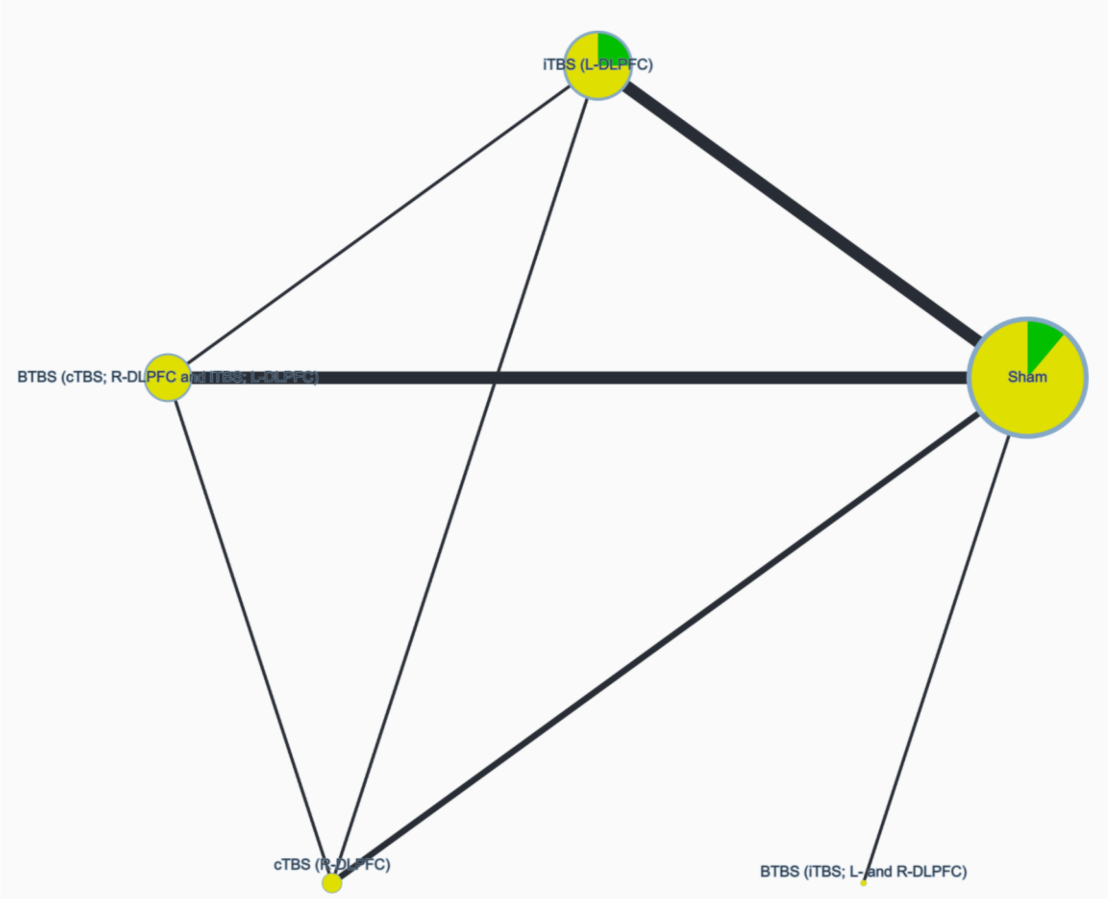

League table (RR with 95% confidence interval)

BTBS (cTBS; R-DLPFC and iTBS; L-DLPFC)	0.650 (0.090, 4.672)	1.110 (0.177, 6.971)	0.361 (0.113, 1.153)	0.731 (0.275, 1.943)
	BTBS (iTBS; L- and R-DLPFC)	1.708 (0.161, 18.131)	0.555 (0.086, 3.577)	1.125 (0.203, 6.239)
		cTBS (R-DLPFC)	0.325 (0.061, 1.740)	0.659 (0.129, 3.350)
			iTBS (L-DLPFC)	2.029 (0.973, 4.232)
				Sham

Global heterogeneity

Between study variance (τ^2): 0.000 (heterogeneity assessment: low)

Random-effects design-by-treatment interaction model

χ 2 statistic: 0.198 (3 degrees of freedom), P value: 0.978

Local heterogeneity (I^2) and incoherence (SIDE test)

	NMA RR	Direct RR	I^2	Indirect RR	P value (SIDE test)
BTBS (cTBS; R-DLPFC and iTBS; L-DLPFC) vs cTBS (R-DLPFC)	1.110 (0.177, 6.971)	1.000 (0.069, 14.553)	na	1.219 (0.098, 15.220)	0.916
BTBS (cTBS; R-DLPFC and iTBS; L-DLPFC) vs iTBS (L-DLPFC)	0.361 (0.113, 1.153)	0.333 (0.039, 2.853)	na	0.372 (0.093, 1.486)	0.932
BTBS (cTBS; R-DLPFC and iTBS; L-DLPFC) vs Sham	0.731 (0.275, 1.943)	0.711 (0.263, 1.923)	0.0%	1.555 (0.009, 259.979)	0.769
cTBS (R-DLPFC) vs iTBS (L-DLPFC)	0.325 (0.061, 1.740)	0.333 (0.039, 2.853)	na	0.311 (0.021, 4.601)	0.969
cTBS (R-DLPFC) vs Sham	0.659 (0.129, 3.350)	0.588 (0.105, 3.299)	0.0%	1.645 (0.012, 222.094)	0.698
iTBS (L-DLPFC) vs Sham	2.029 (0.973, 4.232)	1.988 (0.934, 4.230)	0.0%	2.933 (0.119, 71.981)	0.817

CINeMA confidence rating

Comparison	Number of studies	Within-study bias	Reporting bias	Indirectness	Imprecision	Heterogeneity	Incoherence	Confidence rating
BTBS (cTBS; R-DLPFC and iTBS; L-DLPFC) vs Sham	4	Some concerns	Some concerns	No concerns	Major concerns	No concerns	No concerns	Very low
BTBS (cTBS; R-DLPFC and iTBS; L-DLPFC) vs cTBS (R-DLPFC)	1	Some concerns	Some concerns	No concerns	Major concerns	No concerns	No concerns	Very low
BTBS (cTBS; R-DLPFC and iTBS; L-DLPFC) vs iTBS (L-DLPFC)	1	Some concerns	Some concerns	No concerns	Major concerns	No concerns	No concerns	Very low
BTBS (iTBS; L- and R-DLPFC) vs Sham	1	Some concerns	Some concerns	No concerns	Major concerns	No concerns	No concerns	Very low
cTBS (R-DLPFC) vs Sham	2	Some concerns	Some concerns	No concerns	Major concerns	No concerns	No concerns	Very low
iTBS (L-DLPFC) vs Sham	4	Some concerns	Some concerns	No concerns	Major concerns	No concerns	No concerns	Very low
cTBS (R-DLPFC) vs iTBS (L-DLPFC)	1	Some concerns	Some concerns	No concerns	Major concerns	No concerns	No concerns	Very low
BTBS (cTBS; R-DLPFC and iTBS; L-DLPFC) vs BTBS (iTBS; L- and R-DLPFC)	0	Some concerns	Some concerns	No concerns	Major concerns	No concerns	No concerns	Very low
BTBS (iTBS; L- and R-DLPFC) vs cTBS (R-DLPFC)	0	Some concerns	Some concerns	No concerns	Major concerns	No concerns	No concerns	Very low
BTBS (iTBS; L- and R-DLPFC) vs iTBS (L-DLPFC)	0	Some concerns	Some concerns	No concerns	Major concerns	No concerns	No concerns	Very low
